# Superparamagnetic Fe₃O₄/ZnO/ZnFe₂O₄ nanocomposites for efficient photocatalytic degradation of methylene blue from water under UV light

**DOI:** 10.1038/s41598-025-24533-3

**Published:** 2025-11-18

**Authors:** Alia A. Melegy, Yasser K. Abdel-Monem, Farag A. Ali, Nermine E. Maysour, Ayman M. Atta

**Affiliations:** 1 Department Petroleum Application, Egyptian Petroleum Research Institute, Nasr, 11727 Cairo Egypt; 2 Department Chemistry, Faculty of Science, Menoufia University, Menoufia, Egypt

**Keywords:** Photodegradation, Nanocomposites, Protic poly(ionic liquids), Magnetic, Zinc oxides nanoparticles, Ferrite, Chemistry, Environmental sciences, Materials science, Nanoscience and technology

## Abstract

SA A new series of porous, superparamagnetic nanocomposites was developed by combining Fe₃O₄ and ZnO, as well as ZnFe₂O₄, using a protic poly (ionic liquid) (PIL) as a template. These materials were synthesized at low temperatures, eliminating the need for high-temperature calcination, and were evaluated as photocatalysts for degrading methylene blue (MB), a cationic dye pollutant in water (30 ppm concentration). A linear PIL (LPIL) served as a capping agent to produce well-dispersed ZnO nanoparticles. This ZnO.LPIL was then dispersed during the crosslinking of protic ionic liquid monomer to form a crosslinked PIL (CPIL) nanocomposite matrix. Both the LPIL and CPIL were synthesized via the radical polymerization of triethanolammonium-2-acrylamidomethylpropane sulfonate and triethanolammonium acrylate monomers, with N, N’-methylenebisacrylamide added as a crosslinker for the CPIL. Using an in-situ technique, three types of magnetic nanocomposites (MNCs) were prepared within this matrix: magnetite (Fe₃O₄), zinc ferrite (ZnFe₂O₄), and a core-shell Fe₃O₄@ZnO structure. The chemical composition, crystal structure, and surface morphology of these MNCs were thoroughly characterized. Their thermal, optical, surface, and magnetic properties confirmed their superparamagnetic behavior, porosity, and thermal stability. The photocatalytic performance of these catalysts was assessed for the degradation of MB at pH 12 using a low catalyst dose of 20 mg under light/UV irradiation. The MNCs demonstrated exceptional efficiency and reusability, successfully degrading over 99.4% of the MB dye within 30 min and maintaining this high performance for more than 10 consecutive cycles.

**Supplementary Information:**

The online version contains supplementary material available at 10.1038/s41598-025-24533-3.

## Introduction

The contamination of water resources by industrial pollutants, heavy metals, and organic dyes represents a critical global environmental challenge, posing severe threats to human health, biodiversity, and ecological stability^1,2^. Among these pollutants, methylene blue (MB), a common cationic dye used in textile, paper, and pharmaceutical industries, is particularly concerning. Its release into aquatic ecosystems introduces significant risks due to its inherent toxicity, resistance to biodegradation, and potential carcinogenicity^3^. Consequently, the effective removal of MB from wastewater has become a major research focus, with adsorption and advanced oxidation processes being widely explored^4^. Conventional water treatment methods, including adsorption, coagulation, and membrane filtration, are often hampered by limitations such as low efficiency, high operational costs, the generation of secondary waste, and inadequate performance with complex pollutant mixtures^5^. To overcome these challenges, there is an urgent need for advanced, cost-effective, and sustainable materials capable of efficient and selective pollutant removal. In recent years, magnetic nanocomposites have emerged as promising candidates for water treatment applications. Their appeal lies in their high surface area, ease of separation via an external magnetic field, and tunable surface chemistry^6^. A particularly attractive strategy involves the integration of magnetic nanoparticles with poly (ionic liquids) (PILs). PILs combine the unique properties of ionic liquids—such as high thermal stability, low volatility, and structural versatility—with the mechanical robustness and reusability of a polymeric framework^7–9^. This synergy results in materials with enhanced ionic selectivity, chemical stability, and adsorption capacity. The functional groups on PILs can be tailored to interact specifically with target contaminants, such as organic dyes or heavy metals, through electrostatic, coordination, and hydrophobic interactions, enabling highly efficient and selective extraction^10,11^. Furthermore, the incorporation of magnetic nanoparticles (e.g., Fe₃O₄, MFe₂O₄) into PIL matrices significantly enhances their functionality. This combination facilitates rapid magnetic recovery, minimizing material loss and operational costs^12–15^, while also increasing the number of active sites available for pollutant capture. Despite their promise, the synthesis of tailored PILs can be costly, presenting a barrier to large-scale application^12^. Therefore, developing scalable and economical synthesis routes is crucial for the widespread adoption of PIL-based nanocomposites in water treatment. This work aims to contribute to this effort by designing and evaluating novel magnetic PIL nanocomposites for the efficient degradation and removal of organic pollutants.

Spinel-structured ferrite nanomaterials, such as magnetite (Fe₃O₄), nickel ferrite (NiFe₂O₄), and zinc ferrite (ZnFe₂O₄), have garnered considerable interest for applications in water purification, catalysis, and environmental remediation^14^. Their appeal lies in a combination of favorable properties, including high surface area, excellent chemical stability, and intrinsic magnetism, which enables facile separation and reuse^15^. However, the functional properties of these nanomaterials are often compromised by shortcomings inherent to traditional synthesis methods: such as co-precipitation^16^, sol-gel^17^, hydrothermal^18^, solvothermal^19^, and microemulsion techniques^20^. These methods frequently result in particle agglomeration, wide size distribution, incomplete phase formation, and often require high-temperature calcination. Achieving uniform morphology and phase purity typically necessitates post-synthesis modifications or surfactant-assisted processes, which can introduce impurities or increase production costs. An alternative strategy to enhance practicality is their incorporation into polymeric matrices and composites, which improves mechanical stability and contaminant selectivity^21^. In this context, polyionic liquids (PILs) have emerged as effective media for the synthesis and functionalization of ferrite nanoparticles, offering precise control over their size, morphology, and surface properties^22^. PILs serve as green solvents in solvothermal and hydrothermal synthesis, facilitating the formation of uniform, highly crystalline nanoparticles. They also stabilize particles and prevent aggregation in co-precipitation and enhance the efficiency of microwave-assisted synthesis, enabling rapid and energy-efficient production^23^. Beyond synthesis, PILs are used to functionalize ferrite nanoparticles, introducing specific functional groups (e.g., imidazolium, pyridinium) that significantly enhance their adsorption and catalytic capabilities^24^. PIL-functionalized nanoparticles exhibit a high adsorption capacity for heavy metal ions (e.g., Pb²⁺, Cd²⁺, Cr(VI)) due to the complexation with these functional groups^25^. They are also highly effective at adsorbing organic dyes (e.g., methylene blue (MB), rhodamine B) and degrading them through advanced oxidation processes (AOPs) or photocatalysis^26^. In this respect, PIL-assisted CoFe₂O₄ nanoparticles showed enhanced photocatalytic activity for methyl orange degradation under visible light, attributed to improved charge separation and light absorption^27^. · PIL-modified Fe₃O₄ nanoparticles demonstrated high adsorption capacity for pollutants through electrostatic interactions between the IL cations and anion pollutants^28^. · Similarly, PIL-modified ZnO nanoparticles exhibited high efficiency in the photocatalytic degradation of organic water pollutants^29^. Although Fe₃O₄/ZnO nanocomposites have previously been reported for photocatalytic degradation of MB under visible light irradiation^30^, the present study adopts a distinctly different approach and research aim. Instead of focusing solely on photocatalysis, this work introduces a low-temperature, calcination-free, in-situ synthesis strategy to fabricate ZnO/Fe₃O₄/ZnFe₂O₄–based porous hydrogel nanocomposites. This method integrates nanoparticle nucleation, polymer–nanoparticle interactions, and hierarchical pore generation within a hydrogel framework, resulting in materials with tailored porosity, minimal leaching, and enhanced reusability. Unlike conventional nanoparticle systems, the synthesized hydrogels function as multifunctional adsorbents with strong magnetic separation capability and high structural stability, enabling efficient removal of metal ions and providing a scalable platform for diverse environmental and industrial applications. Thus, while the earlier work demonstrates the photocatalytic potential of Fe₃O₄/ZnO composites^30^, this work expands the scope by developing structurally engineered, reusable, and multifunctional nanocomposites that address broader water purification challenges. Moreover, this work presents a systematic investigation into the use of ferrite nanomaterials for water treatment, with a focus on their synthesis, structural and magnetic characterization, and application efficiency. The study aims to elucidate the mechanisms governing pollutant removal and assess the potential of these nanomaterials for scalable, sustainable water purification technologies. Poly (ionic liquids) (PILs) offer a versatile platform for nanomaterial synthesis, enabling precise control over particle size, morphology, and surface properties. PILs have been used to synthesize metal oxide nanoparticles (e.g., TiO₂, ZnO, and Fe₃O₄) with controlled crystallinity and high surface area, which are critical for adsorption and photocatalytic applications. A key objective of this study is the preparation of porous nanocomposites via an in-situ technique using linear (LPIL) and crosslinked protic poly (ionic liquids) (CPIL) based on triethanolammonium-2-acrylamidomethylpropane sulfonate-co-triethanolammonium acrylate as a template, with zinc oxide or ferrite nanoparticles. These nanocomposites are designed to serve as photocatalysts for the removal of MB dye from contaminated water under both visible and UV light irradiations. The choice between LPIL and CPIL as a template for Fe₃O₄/ZnO/ZnFe₂O₄ and Fe₃O₄@ZnO nanocomposites significantly influences photocatalytic performance due to differences in structural stability, ionic conductivity, and interfacial interactions. LPILs, with their flexible polymeric chains, enhance charge transport and provide a homogeneous dispersion of metal oxide nanoparticles, thereby improving light absorption and reducing electron-hole recombination rates. However, their solubility and mechanical instability under prolonged irradiation can limit recyclability. In contrast, CPILs form rigid, three-dimensional networks that stabilize the nanocomposite structure, preventing nanoparticle aggregation and leaching during photocatalytic cycles. This crosslinking enhances thermal and chemical stability while maintaining sufficient ionic mobility for efficient charge separation. The porous architecture of CPILs can also facilitate better reactant diffusion, further boosting photocatalytic efficiency. Thus, while LPILs may offer superior initial activity, CPILs provide enhanced durability and reusability, making them more suitable for long-term applications in environmental remediation. MB dye was selected for this study due to its well-established degradation pathway and widespread use as a benchmark compound in photocatalytic testing. We fully acknowledge that different pollutants (e.g., anionic dyes, cationic dyes, or complex organic compounds) may exhibit varying interactions with the Fe₃O₄/ZnO/ZnFe₂O₄ and PIL composite. The use of MB allowed for a systematic evaluation of the fundamental photocatalytic performance under controlled conditions, particularly in light of the unique electron-transfer and surface interactions facilitated by the ionic liquid polymer-modified nanocomposite system.

## Experimental

### Materials

The following high-purity chemicals were procured from Sigma-Aldrich Chemicals Co. and utilized directly without additional purification: methylene blue (MB), acrylic acid (AA), 2-acrylamido-2-methylpropane sulfonic acid (AMPS), N,N-methylenebisacrylamide (MBA), triethanolamine (TEA) and ammonium persulfate (APS). Zinc nitrate hexahydrate (Zn(NO_3_)_2_.6H_2_O), NaOH, anhydrous ferric chloride (FeCl_3_), potassium iodide (KI), and ammonium hydroxide (25%) were used to prepare zinc oxide, zinc ferrite and magnetite nanocomposites. Ethylenediaminetetraacetic acid (EDTA), isopropanol and silver nitrate were used as scavengers.

### Preparation technique

#### Synthesis of LPIL and CPIL

The solvent-free copolymerization of ionic liquid monomers was carried out under a nitrogen atmosphere. Acidic monomers, AMPS or AA (0.1 mol), were quaternized with triethanolamine (TEA, 0.1 mol) as previously reported^31^. The mixture was stirred continuously at 278 K for 24 h, producing clear amber-colored QAMPS or QAA monomers. Subsequently, 0.6 wt% ammonium persulfate (APS, relative to QAMPS) was added to an equimolar blend of QAA (0.1 mol each). Polymerization was initiated at 278 K and the temperature gradually raised to 333 K over 18 h, yielding viscous solutions that were vacuum-dried to obtain transparent amber oils. The reaction yield of QAMPS/QAA was 98.2%, and the product was designated as LPIL. For CPIL synthesis, the procedure was identical to that of LPIL, except that N, N′-methylenebisacrylamide (MBA, 1 wt% of total monomer mass) was introduced during polymerization. The resulting crosslinked material was obtained as solid rods and post-heated overnight at 378 K.

#### Synthesis of ZnO.LPIL and ZnO.CPIL nanocomposites

ZnO nanoparticles capped with LPIL were prepared following our previous method^31^. Briefly, Zn(NO₃)₂·6 H₂O (0.1 mol) was dissolved in 50 mL of distilled water and mixed with LPIL (4 g) under vigorous stirring to obtain a clear solution. Sodium hydroxide (0.2 M in ethanol, 30 mL) was added dropwise with vigorous stirring until the solution pH reached 12.5. The mixture was heated at 318 K (45 °C) for 4 h, producing a milky suspension. After cooling, the suspension was centrifuged at 15,000 rpm for 10 min, and the precipitate was washed several times with water and ethanol to remove impurities. The obtained white solid was dried at 383 K for 5 h to yield ZnO.LPIL nanoparticles. For composite preparation, AMPS (0.1 mol) or QAMPS and AA or QAA (0.1 mol) were dissolved in 10 mL of water with MBA (1 wt% relative to monomers). Different loadings of ZnO.LPIL (1–5 wt% relative to monomers) were dispersed into the solution under nitrogen atmosphere at room temperature. APS initiator (0.01 mol%) dissolved in 5 mL of water was introduced after purging with nitrogen for 15 min. The mixture was heated to 333 K until solid polymer composites formed. The ZnO.CPIL and related composites were post-cured at 378 K for 24 h, followed by water rinsing for 24 h to remove soluble by-products.

#### In-situ synthesis of magnetic nanocomposites (MNCs)

Magnetic nanocomposites (MNCs) based on Fe₃O₄.CPIL, ZnFe₂O₄.CPIL, and Fe₃O₄@ZnO.CPIL were synthesized via an in-situ technique. Preparation of ZnFe₂O₄.CPIL: Solution A was prepared by dissolving FeCl₃ (40 g, 0.24 mol) and Zn(NO₃)₂·6 H₂O (0.12 mol) in 400 mL of distilled water. Solution B contained KI (13.2 g, 0.12 mol) in 50 mL of water. The two solutions were combined at room temperature under continuous nitrogen purging and stirred for 1 h. The resulting precipitate was filtered, while the filtrate and washings were absorbed into CPIL powder to form a gel. Hydrolysis was initiated by dropwise addition of 200 mL of 25% ammonia solution under nitrogen protection, followed by heating at 323 K. A brownish-black ZnFe₂O₄.CPIL precipitate was obtained after 4 h of stirring. The product was filtered, washed thoroughly with water, and vacuum-dried at 303 K to avoid thermal degradation. The same procedure was followed without Zn(NO₃)₂, while the KI concentration was adjusted to 0.08 mol used to prepare of Fe₃O.CPIL. Preparation of Fe₃O₄@ZnO.CPIL composite was synthesized as above, but ZnO.CPIL was used in place of CPIL. Preparation of ZnFe₂O₄.AMPS/AA and Fe₃O₄.AMPS/AA composites were prepared by the same procedure, except that crosslinked AMPS/AA polymers were used instead of CPIL.

### Characterization of synthesized ILs

Fourier-transform infrared spectroscopy (FT-IR, Perkin Elmer, USA in the wave number range of 4000–400 cm⁻¹) was employed to analyze the stretching vibration frequencies of the synthesized composites. The optical properties were investigated using a JASCO V-750 UV–Vis spectrophotometer by recording reflectance spectra at varying wavelengths, and the optical band gap (E₉) was estimated from Tauc’s plots. Additional absorption spectra were recorded on a PerkinElmer Lambda 35 UV–Vis spectrophotometer after dispersing the powdered samples in distilled water under vigorous stirring to ensure homogeneous suspensions. Photoluminescence (PL) spectra were obtained on a Perkin Elmer LS 55 spectrofluorometer using an excitation wavelength of 320 nm. The pore size distribution was determined using the Barrett–Joyner–Halenda (BJH) method, while the specific surface area and pore volume were calculated by the Brunauer–Emmett–Teller (BET) technique. N₂ adsorption–desorption isotherms were recorded at 77 K with an automated sorption analyzer (Micromeritics ASAP 2000) after degassing the samples at 150 °C overnight under nitrogen flow. Methylene blue (MB) dye concentrations were determined with a double-beam UV–Vis spectrophotometer (Shimadzu UV-1208) at λmax = 662 nm using a 10 mm quartz cuvette. Aliquots (5 mL) were withdrawn at regular time intervals for spectral measurements. The morphology of ZnO nanoparticles was examined by transmission electron microscopy (TEM, JEOL JEM-2100 F, 200 kV), while surface composition and particle size were analyzed by scanning electron microscopy (SEM, JEOL JXA-840 A, 20 kV). Elemental composition was determined by energy-dispersive X-ray spectroscopy (EDX). The crystalline phase and average crystallite size were studied using X-ray diffraction (XRD, Bruker D2 powder diffractometer, 30 kV, 10 mA, Cu Kα radiation, λ = 0.15406 nm) at 25 °C. Thermal stability was evaluated by thermogravimetric and differential thermogravimetric analysis (TGA-DTG, Shimadzu TGA-50) under nitrogen atmosphere. Approximately 10 mg of sample was heated from room temperature to 800 °C at 10 °C/min. Magnetic properties were assessed using a vibrating sample magnetometer (VSM, LakeShore). Finally, inductively coupled plasma optical emission spectroscopy (ICP-OES, Agilent 5800, USA) was used to examine catalyst leaching in water during photodegradation.

### Photocatalytic activity and scavenging studies for photodegradation of MB from water

For photocatalytic experiments, 10–100 mg of the nanocomposite catalyst was dispersed in 100 mL of aqueous methylene blue (MB, 30 ppm) solution to form a homogeneous suspension. The mixture was first kept in the dark for 30 min under continuous stirring in a cylindrical photoreactor to establish adsorption–desorption equilibrium between the dye molecules and the photocatalyst surface. After equilibration, the suspension was irradiated with a 150 W UV lamp for a maximum of 120 min. At regular intervals of 10 min, aliquots were withdrawn, centrifuged to separate the catalyst, and analyzed using a UV–Vis spectrophotometer. The degradation efficiency was calculated using the equation: Degradation (%) = [(Abs_o_-Abs_t_)/Abs_o_] x 100, where Abs_o_ denotes the initial absorbance of the dye and at time t, respectively. To investigate the role of reactive oxygen species (ROS) in the photocatalytic degradation mechanism, scavenger experiments were carried out under visible light illumination from a 500 W halogen lamp positioned 10 cm above the reactor (4 cm diameter). Specific scavengers were introduced into the MB solution: EDTA (5 mM; 0.186 g in 100 mL) for photogenerated holes (h⁺), isopropyl alcohol (2% v/v; 2 mL in 100 mL) for hydroxyl radicals (·OH), and silver nitrate (0.5 mM; 0.0085 g in 100 mL) for photogenerated electrons (e⁻). In each experiment, 20–30 mg of photocatalyst was added to 50 mL of MB solution containing the respective scavenger for 2 h. At defined time intervals, aliquots were collected, centrifuged, and analyzed using UV–Vis spectroscopy. The decrease in the absorption peak intensity at λ = 595 nm was used to monitor MB degradation in the presence of scavengers.

## Results and discussion

### Reaction conditions of MNCs

**Scheme 1:**
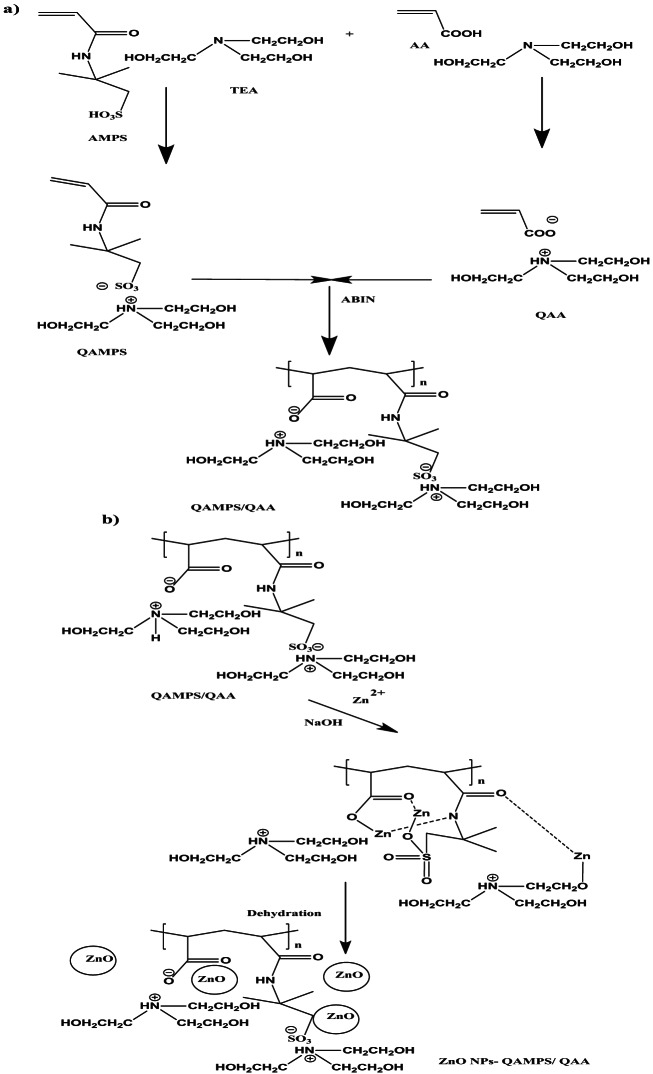
Synthesis route of (**a**) ZnO.LPIL and (**b**) ZnO.CPIL.

**Scheme 2:**
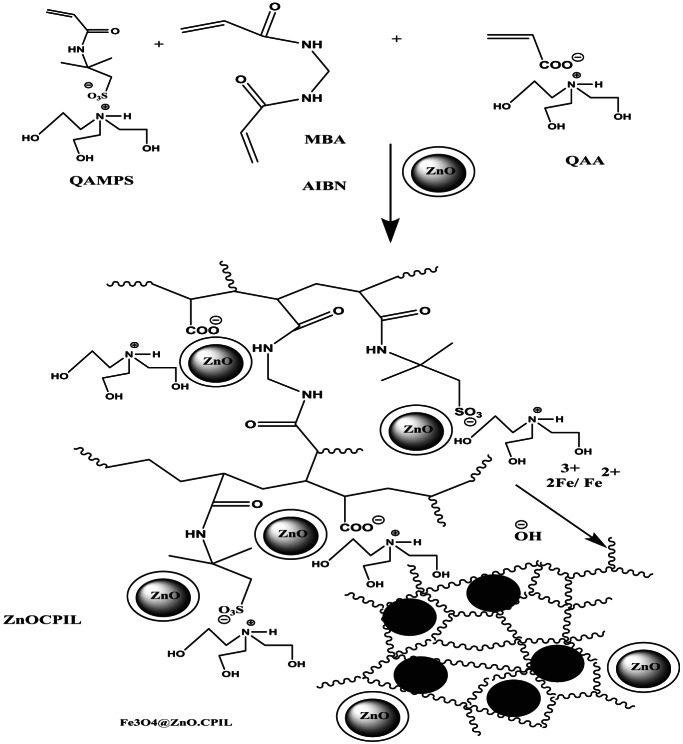
Synthesis route of Fe_3_O_4_@ZnO.CPIL nanocomposites.

The development of magnetic nanocomposites (MNCs) has attracted considerable interest due to their wide-ranging applications in environmental remediation, biomedical engineering, and catalysis. Among these, hydrogel-based magnetic nanocomposites are particularly appealing because they integrate the high surface area, hydrophilicity, and tunable porosity of hydrogels with the magnetic responsiveness of embedded nanoparticles. A major challenge in fabricating such systems, however, lies in preventing the leaching of magnetic nanoparticles (MNPs) from the hydrogel matrix. Nanoparticle leakage not only diminishes the functional performance of the composites but also raises serious concerns regarding environmental contamination and nanoparticle-related toxicity. Traditional *ex-situ* fabrication methods, which rely on the physical incorporation of pre-synthesized MNPs into the hydrogel network, often suffer from weak particle–matrix interactions and poor dispersion. As a result, MNPs may detach under mechanical stress or pH fluctuations. To address these limitations, in-situ synthesis has emerged as a more robust strategy, whereby nanoparticles are nucleated and grown directly within the hydrogel network. This approach ensures stronger chemical interactions, minimizes leaching, and significantly improves the structural integrity of the composite. In this study, we introduce a novel in-situ approach that exploits the hydrogel’s microporous structure and reactive functional groups to confine nanoparticle growth, resulting in magnetite-loaded hydrogel nanocomposites with exceptional stability under harsh operating conditions. These composites were subsequently evaluated for their magnetic properties, leaching resistance, and potential for targeted removal of water pollutants. In parallel, we aim to develop a porous hydrogel nanocomposite by incorporating ZnO nanoparticles during hydrogel crosslinking copolymerization, yielding a stable hydrogel foam with enhanced absorption capacity. This material was employed for the efficient adsorption of Fe²⁺/Fe³⁺ cations from aqueous solutions, followed by in-situ hydrolysis to generate porous metal oxide–loaded hydrogel nanocomposites. Conventionally, ZnO nanoparticle synthesis requires high-temperature calcination (≥ 673 K) of zinc hydroxide or zinc carbonate precursors to obtain crystalline ZnO phases. By contrast, the present work demonstrates a low-temperature (< 373 K), calcination-free route for producing well-dispersed ZnO nanoparticles using LPIL templates derived from tris(2-hydroxyethyl)−2-acrylamido-2-methylpropane sulfonate-co-tris(2-hydroxyethyl)acrylate (QAMPS/QAA), as illustrated in Scheme 1a–b. In this system, QAMPS/QAA functions dually as a chelating agent and as a nanoreactor. The sulfonate (-SO₃H) and hydroxyl groups coordinate with Zn²⁺ ions, forming a stable zinc–LPIL complex. This complexation confines Zn(OH)₂ nucleation within the LPIL network, suppressing uncontrolled aggregation. Moreover, the protic functional groups (-SO₃H and quaternary -N⁺) act as catalysts for Zn(OH)₂ dehydration, enabling the formation of ZnO nuclei at temperatures below 100 °C without the need for calcination. As shown in Scheme 1b, this strategy yields well-dispersed ZnO nanoparticles confined within the polymeric matrix, leading to enhanced stability, improved porosity, and superior performance of the resulting hydrogel nanocomposites. The crosslinking copolymerization of QAMPS/QAA using MBA (1 wt%) as the crosslinker and ZnO nanoparticles (ZnO NPs) at concentrations ranging from 1 to 5 wt% (Scheme 1a–b) is illustrated in Scheme 2. The in-situ development of magnetic nanocomposite (MNC) porous hydrogels through swelling in iron cations (Fe²⁺/Fe³⁺), followed by hydrolysis with ammonia solution, represents a novel and efficient strategy for fabricating high-performance adsorbents for water purification. Post-curing experiments revealed that hydrogel samples treated at temperatures above 100 °C and containing more than 2 wt% ZnO NPs exhibited pronounced foaming behavior. This phenomenon can be attributed to three main factors. First, at ZnO concentrations exceeding 2 wt%, the nanoparticles form interconnected structures that substantially increase the viscosity of the reaction medium. Elevated viscosity impedes bubble coalescence and escape, while the resulting viscoelastic interfaces stabilize entrapped gas bubbles and hinder mass transfer of dissolved gases. Second, the high surface area of ZnO NPs accelerates the decomposition of quaternary ammonium groups (via Hofmann elimination), enhances sulfonate group degradation, and catalyzes water-splitting reactions at the nanoparticle surface. These processes generate gaseous byproducts (NH₃, CO₂, SO₂) that act as nucleation sites for bubble formation. Third, high ZnO loadings promote a Pickering emulsion–like stabilization of bubbles through electrostatic interactions, nanoparticle surface charges, and steric hindrance provided by polymeric capping agents. The ionic nature of the AMPS and AA monomers further enhances the dispersion of ZnO NPs and increases interfacial activity, while the zwitterionic character of the growing polymer matrix strengthens nanoparticle–polymer interactions. The resulting foamed ZnO–QAMPS/QAA hydrogel precursor, comprising a crosslinked polyelectrolyte network (e.g., acrylamide, acrylic acid, and sulfonated polymers), is subsequently swollen in Fe²⁺/Fe³⁺ ion solutions. Electrostatic interactions between negatively charged functional groups (e.g., carboxylate, sulfonate) and the positively charged iron ions facilitate uniform incorporation of iron species into the hydrogel matrix (Scheme 2). Upon treatment with ammonia solution (NH₄OH), the alkaline environment induces the nucleation and growth of iron hydroxide nanoparticles within the hydrogel, which act as porogens and generate voids, thereby enhancing porosity. Concurrently, partial oxidation of Fe²⁺ in the presence of Fe³⁺ drives the formation of magnetic Fe₃O₄@ZnO nanoparticles and zinc ferrite (ZnFe₂O₄) through the following reactions (1–2):

1 $$\text{Fe}^{2+}+\text{2Fe}^{3+}+\text{8OH}^-\Rightarrow\text{Fe}_3\text{O}_4+\text{4H}_2\text{O}$$

2 $$\text{Zn}^{2+}+\text{2Fe}^{3+}+\text{8OH}^-\Rightarrow\text{ZnFe}_2\text{O}_4+\text{4H}_2\text{O}$$

The ammonia treatment not only generates Fe₃O₄ but also induces porous structure due to gas evolution (NH₃ decomposition, H₂O release), mesopores (2–50 nm) from iron hydroxide nanoparticle aggregation and micropores (< 2 nm) within the polymer network itself.

This mechanistic pathway highlights the structural evolution of ZnO-loaded porous hydrogels into multifunctional MNCs with strong stability, high porosity, and superior adsorption performance. The synergistic roles of ZnO NPs, ionic monomers, and in-situ iron hydroxide formation provide distinct advantages over conventional hydrogels and powdered adsorbents for advanced water purification applications. The synthesized porous MNCs will be systematically characterized to evaluate their structural, morphological, and surface properties, with particular emphasis on adsorption efficiency, reusability, and multi-functionality. By combining in-situ nanoparticle formation, polymer–nanoparticle interactions, and controlled pore generation, this work establishes a sustainable and scalable strategy for designing advanced nanocomposites with tailored porosity and enhanced reactivity. Such materials hold significant promise for environmental and industrial applications, particularly in water purification, catalysis, and magnetic separation technologies.

### Chemical structures and surface morphologies

**Fig. 1:**
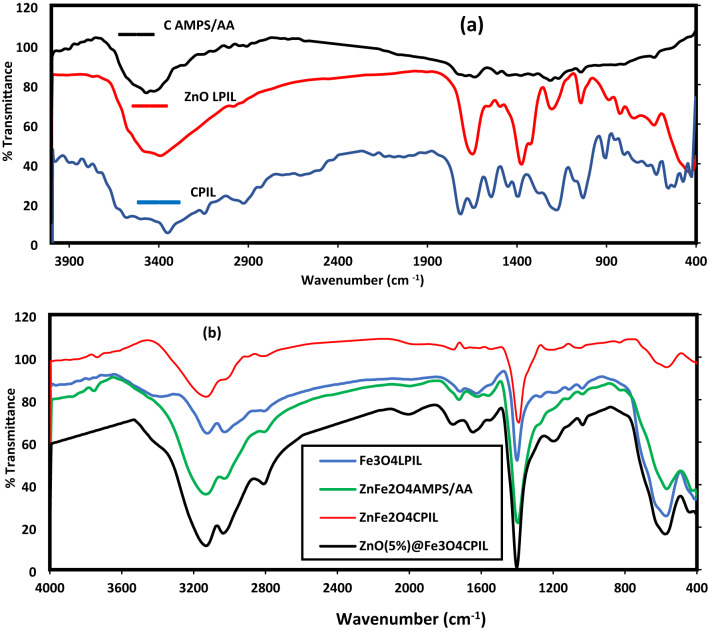
The FTIR spectra of (**a**) CPIL, CAMPS/AA with ZnO.LPIL and (**b**) MNCs.

FTIR spectra were used to confirm the capping of ZnO nanoparticles with LPIL (QAMPS/QAA) and to study the interactions between the polymer and nanoparticle surface (Fig. 1a, b)^32,33^. FTIR band assignments of CPIL, ZnO.LPIL and MNCs were represented in Table 1. The FTIR spectrum of the CPIL, CAMPS/AA and ZnO.LPIL were summarized in Fig. 1a. The FTIR spectra of MNCs based on Fe_3_O_4_.CPIL, Fe_3_O_4_.AMPS/AA, ZnFe_2_O_4_.CPIL and Fe_3_O_4_@ZnO.CPIL were collected in Fig. 1b. In Fig. 1a, the spectra of CPIL, CAMPS/AA, and ZnO.LPIL exhibit characteristic bands from hydrophilic and anionic groups. The broad band near 3400 cm⁻¹ corresponds to O–H stretching of hydroxyl groups from TEA in QAMPS and QAA, confirming the hydrophilic nature of the copolymer. Bands at 1723 and 1649 cm⁻¹ represent C = O stretching of QAA and QAMPS units. Sulfonate (–SO₃⁻) vibrations appear at 1186 and 1039 cm⁻¹, verifying the anionic character. Upon ZnO capping, the O–H band broadened, suggesting hydrogen bonding with the ZnO surface. The C = O bands merged into a broad feature (1740–1630 cm⁻¹), indicating coordination between carbonyl oxygen and Zn²⁺ ions. Sulfonate peaks weakened, consistent with their role in electrostatic stabilization. Importantly, the Zn–O stretching band at 467 cm⁻¹ confirmed preservation of the ZnO core. New combined bands at 1648 and 1375 cm⁻¹ supported chemical grafting of ZnO with the copolymer. Additional COO⁻ stretching bands (1580 and 1400 cm⁻¹) indicated decarboxylation of AA units, while reduced sulfonate intensity suggested partial AMPS decomposition. In Fig. 1b, FTIR spectra of MNCs based on AMPS/AA and QAMPS/QAA hydrogels confirmed in-situ formation of ZnO, Fe₃O₄, and ZnFe₂O₄. Characteristic Fe–O (550 cm⁻¹, tetrahedral) and Zn–O (430 cm⁻¹, octahedral) vibrations verified spinel ZnFe₂O₄ formation. The FTIR data support a foaming mechanism where the capped ZnO NPs act as nanoscale heating centers due to their high thermal conductivity. The FTIR spectra of MNCs based on AMPS/AA and QAMPS/QAA hydrogels (Fig. 1b) were used to confirm formation of ZnO and Fe_3_O_4_ or zinc ferrite ZnFe_2_O_4_ based on in-situ technique. The metal-oxygen vibration region (400–600 cm⁻¹) showed a characteristic splitting pattern at 550 cm⁻¹ (Fe-O tetrahedral) and 430 cm⁻¹ (Zn-O octahedral), confirming spinel formation of ZnFe_2_O_4_^34^. The sulfonate group vibrations (1180 cm⁻¹ and 1045 cm⁻¹) (Fig. 1b) exhibited stronger attenuation than in ZnO systems (Fig. 1a), suggesting enhanced metal-sulfonate coordination.

**Table 1:** FTIR band assignments of CPIL, ZnO.LPIL and MNCs.

Composites	Observation in composites	Assignment	Wave number (cm^−1^)
CPIL, ZnO.LPIL and MNCs	Broad band with ZnO (H-bonding)	O-H stretching	3400
CPIL, ZnO.LPIL and MNCs	Strong shifted/meged from 1740 to 1630 cm^−1^	C = O stretching	1723, 1649
CPIL, ZnO.LPIL and MNCs	Decarboxylation of AA units	COO- stretching	1580, 1400
CPIL, ZnO.LPIL and MNCs	Medium bands observed in all composites	C-H bending	1450 − 1380
CPIL, ZnO.LPIL and MNCs	Reduced intensity, metal coordination	S = O stretching	1186, 1039–1045
MNCs	Strong broad present in MNCs	Fe-O stretching	550–580
ZnMNCs	present in MNCs contain Zn	Zn-O	430

**Fig. 2:**
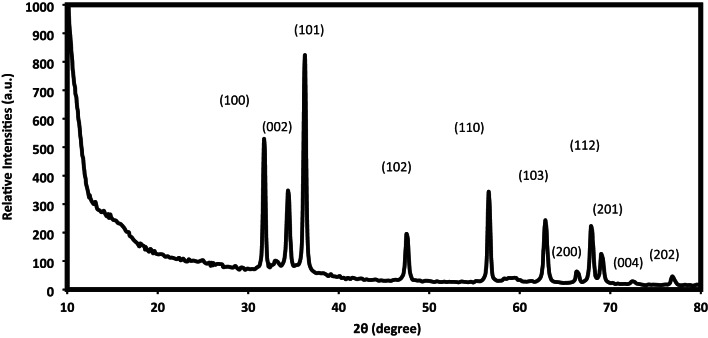
XRD diffractogram of ZnO. LPIL nanocomposite.

**Fig. 3:**
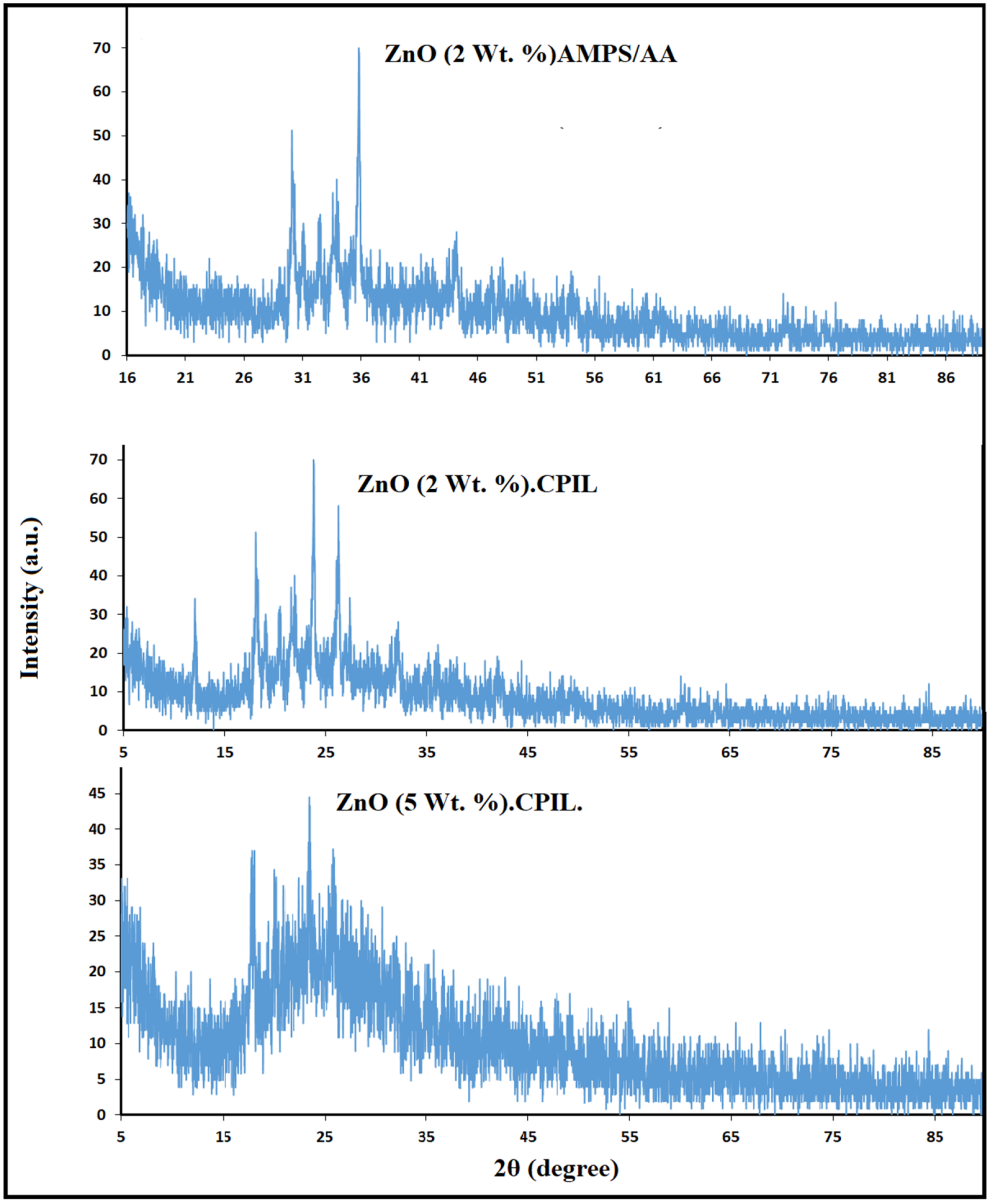
XRD diffractograms of ZnO composites with AMPS/AA and CPIL.

XRD analysis was carried out to examine the composition, crystallinity, and structural interactions of ZnO, Fe₃O₄, and Fe₃O₄@ZnO nanoparticles within LPIL, CPIL, and crosslinked AMPS/AA polymer matrices from their diffractograms represented in Figs. 2 and 3. The diffraction peaks and their Miller indices (hkl) of ZnO.LPIL and b) MNCs were summarized in supplementary file in **Tables S1 to S6**. The diffraction pattern of ZnO.LPIL (Fig. 2) shows sharp and intense peaks at 2θ values corresponding to the (100), (002), (101), (102), (110), (103), (200), (112), (201), (004), and (202) planes, confirming the formation of a hexagonal wurtzite structure (JCPDS Card No. 36–1451)^35^. The sharpness and high intensity of these peaks indicate well-ordered crystalline domains and a high degree of crystallinity. In contrast, the diffractogram of ZnO (2 wt%) incorporated into AMPS/AA (Fig. 3) displays a broad amorphous hump between 20° and 40°, with only weak ZnO peaks visible. This suggests that ZnO is either weakly crystalline or highly dispersed within the AMPS/AA network. The suppressed diffraction intensity and elevated baseline signal reflect strong interactions between ZnO and the polymer matrix, which reduce particle aggregation and partially disrupt long-range crystalline order. For ZnO incorporated into CPIL (Fig. 3), diffraction patterns reveal moderate-intensity peaks with noticeable broadening at 2 wt%, indicating partial retention of ZnO crystallinity in a nano-sized or well-dispersed state. At higher loading (5 wt%), the peaks become more distinguishable, particularly at the (100) and (101) planes, reflecting improved crystalline ordering. This enhancement suggests that at higher concentrations ZnO undergoes partial aggregation or domain formation, which restores some of its intrinsic crystalline structure. Overall, the XRD results demonstrate that the polymer environment significantly influences the crystallinity of ZnO nanoparticles. AMPS/AA strongly disrupts long-range order, whereas CPIL matrices allow partial crystallinity to be preserved, with higher ZnO loading (5 wt%) enhancing detectable diffraction planes. Such structural variations are critical for tailoring the optical, electronic, and catalytic performance of ZnO–PIL hybrid nanocomposites.

**Fig. 4:**
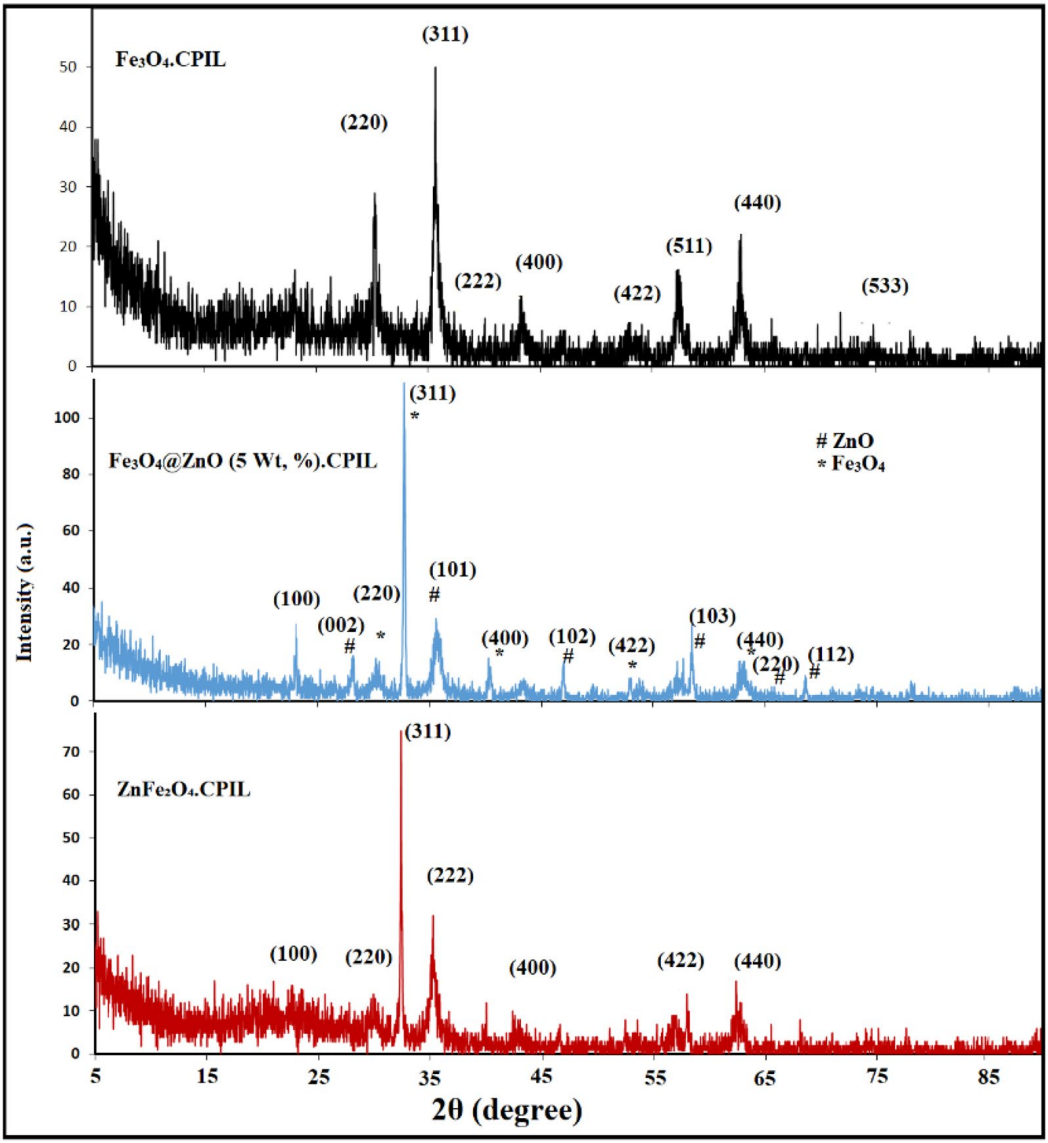
XRD diffractograms of (**a**) Fe_3_O_4_.CPIL, (**b**) Fe_3_O_4_@ZnO (5 Wt, %). CPIL and (**c**) ZnFe₂O₄.CPIL nanocomposites.

The crystal structure and phase purity of magnetite (Fe₃O₄), Fe₃O₄@ZnO, and ZnFe₂O₄ nanocomposites embedded in CPIL were investigated by XRD (Fig. 4). All samples exhibited characteristic diffraction peaks of Fe₃O₄ at 2θ ≈ 18.3°, 30.2°, 35.5°, 43.2°, 53.5°, 57.1°, and 62.7°, corresponding to the (111), (220), (311), (400), (422), (511), and (440) planes (JCPDS No. 19–0629). The dominant (311) reflection confirmed the spinel structure and high crystallinity of magnetite, with no secondary phases such as hematite (α-Fe₂O₃) or maghemite (γ-Fe₂O₃) detected, indicating excellent phase purity^36^. For Fe₃O₄@ZnO composites, the 2 wt% ZnO sample showed well-defined ferrite peaks, while the 5 wt% ZnO composite displayed sharper and more intense reflections, especially at (311) and (440), suggesting crystallite growth and partial restoration of long-range order. However, excessive ZnO incorporation also appeared to reduce ferrite crystallinity, possibly due to the formation of amorphous Zn-rich domains. In ZnFe₂O₄.CPIL, diffraction peaks corresponding to (220), (311), (400), (422), (511), and (440) planes were observed, consistent with a spinel zinc ferrite structure, with the intense (311) peak confirming successful Zn²⁺ substitution into the ferrite lattice^37^. The broad peak at lower angles may be attributed to the amorphous nature of the CPIL matrix, which does not contribute significantly to crystalline diffraction. This amorphous hump also confirms the successful encapsulation or embedding of Fe₃O₄ nanoparticles within the organic matrix, where the CPIL may restrict crystal growth and induce a degree of structural disorder at the interface. Finally, it can be concluded that CPIL network likely plays a role in stabilizing the nanoparticles and preventing agglomeration, which helps retain their nanocrystalline nature. Moreover, the CPIL matrix may influence the surface structure and stress state of the embedded Fe₃O₄, leading to slight peak broadening or shifting. Crystallite Size Estimation Using the Debye–Scherrer equation, the average crystallite size D of the Fe₃O₄ nanoparticles can be estimated from the full width at half maximum (FWHM) of the (311) peak: D = K λ/β cos θ; where D, K, λ, β and θ are crystallite size, shape factor (typically 0.9), X-ray wavelength (e.g., 1.5406 Å for Cu Kα), FWHM in radians and Bragg angle, respectively (Tables S1-S6). Crystallite size calculations using the Debye–Scherrer equation revealed values of 42.4 nm for ZnO.LPIL, 70.1 nm for Fe₃O₄@ZnO (2 wt%) CPIL, 52.6 nm for Fe₃O₄@ZnO (5 wt%) CPIL, and 140.2 nm for Fe₃O₄.CPIL. The corresponding d-spacing values were 2.477 Å, 2.732 Å, 2.733 Å, and 2.766 Å, respectively. These results demonstrate that ZnO loading strongly influences crystallinity, crystallite size, and structural order, while the CPIL matrix plays a key role in nanoparticle stabilization and phase integrity.

### Surface morphologies thermal and magnetic properties

**Fig. 5:**
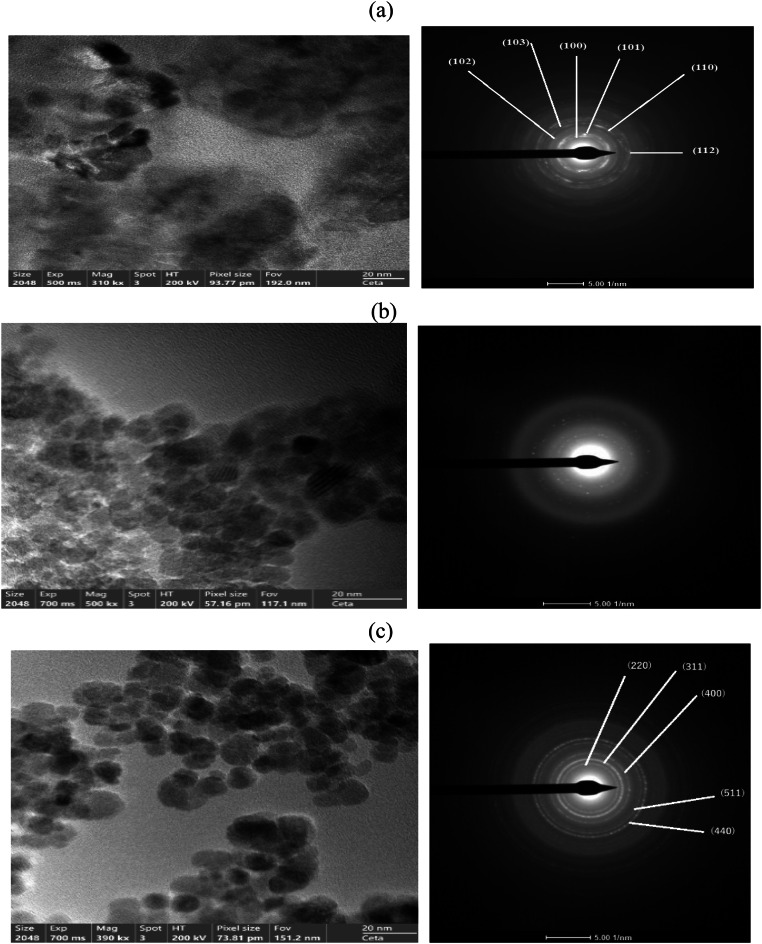
TEM and SAED micrographs of (**a**) ZnO.LPIL, (**b**) Fe_3_O_4_@ZnO (2 Wt. %).CPIL and (**c**) Fe_3_O_4_@ZnO (5 Wt. %).CPIL.

The XRD patterns confirmed the crystalline structure of ZnO nanoparticles, characterized by the hexagonal wurtzite phase, and the successful formation of spinel ZnFe₂O₄ in Fe₃O₄@ZnO.CPIL composites. The absence of separate peaks for Fe₃O₄ and ZnO in the nanocomposites indicated the formation of a single-phase spinel structure, corroborating the strong interaction between metal oxides and the CPIL matrix. TEM imaging and SAED analysis were performed and represented in Fig. 5a–c to investigate the morphology and microstructure of ZnO.LPIL nanoparticles and their incorporation into CPIL matrices with 2 and 5 wt% ZnO, followed by in-situ synthesis of Fe₃O₄@ZnO nanocomposites. The ZnO.LPIL sample (Fig. 5a) shows quasi-spherical to rod-like nanoparticles with lattice fringes consistent with the (100), (002), and (101) planes of hexagonal wurtzite ZnO, confirming their crystalline nature. The SAED pattern displays sharp concentric rings, characteristic of polycrystalline materials composed of randomly oriented nanocrystallites, in agreement with the XRD results. A lighter amorphous halo around many particles indicates the presence of the LPIL shell, which stabilizes the nanoparticles through electrostatic and steric interactions, preventing agglomeration and promoting uniform dispersion. The ZnO.LPIL morphology (Fig. 5a) exhibits a quasi-spherical to rod-like morphology with varying contrast, indicating differences in local density or orientation. These reflections are consistent with previously reported diffraction data for ZnO nanoparticles synthesized by wet chemical and solvothermal methods^38^. Several domains show visible lattice fringes with clear periodicity, which confirms the crystalline nature of the ZnO core. The measured interplanar spacings from the high-resolution lattice fringes (not explicitly measured here but visible) are consistent with the (100), (002), and (101) planes of the hexagonal wurtzite ZnO phase, as also supported by SAED patterns (Fig. 5a). Surrounding many of the crystalline domains is a lighter, less-contrasted amorphous halo, indicative of the LPIL coating. The LPIL appears to form a semi-continuous shell around the nanoparticles or aggregates, likely through electrostatic interactions and hydrogen bonding. This shell is not crystalline, consistent with the behavior of PILs in similar hybrid systems^39^. The LPIL acts as both a steric and electrostatic stabilizer, preventing particle agglomeration and enabling better dispersion of the ZnO nanoparticles. For Fe₃O₄@ZnO nanocomposites with 2 wt% ZnO (Fig. 5b), TEM images reveal well-dispersed, near-spherical nanoparticles embedded in the CPIL matrix, with sizes mostly below 20 nm. The SAED pattern (Fig. 5b) shows sharp concentric rings, confirming a polycrystalline structure and high crystallinity, consistent with the ferrite spinel phase. At higher ZnO loading (5 wt%, Fig. 5c), nanoparticles appear denser and more agglomerated, forming larger clusters. The corresponding SAED rings remain visible and concentric but exhibit stronger intensity and slight broadening, suggesting overlapping domains and reduced dispersion while maintaining overall crystallinity. The diffraction rings in both composites match the cubic spinel structure of ZnFe₂O₄ (Fd3̅m), with reflections indexed to the (220), (311), (400), (511), and (440) planes^40^. No separate ZnO or Fe₃O₄ rings were observed, supporting the formation of a single-phase ZnFe₂O₄ rather than a core–shell Fe₃O₄@ZnO system. Together with XRD, these results confirm that at low loading (2 wt%), nanoparticles are better dispersed within the CPIL matrix, while higher loading (5 wt%) promotes aggregation without compromising crystallinity. The successful formation of ZnFe₂O₄ within the polymeric network highlights the potential of these nanocomposites for multifunctional applications in catalysis, magnetic separation, and environmental remediation.

**Fig. 6:**
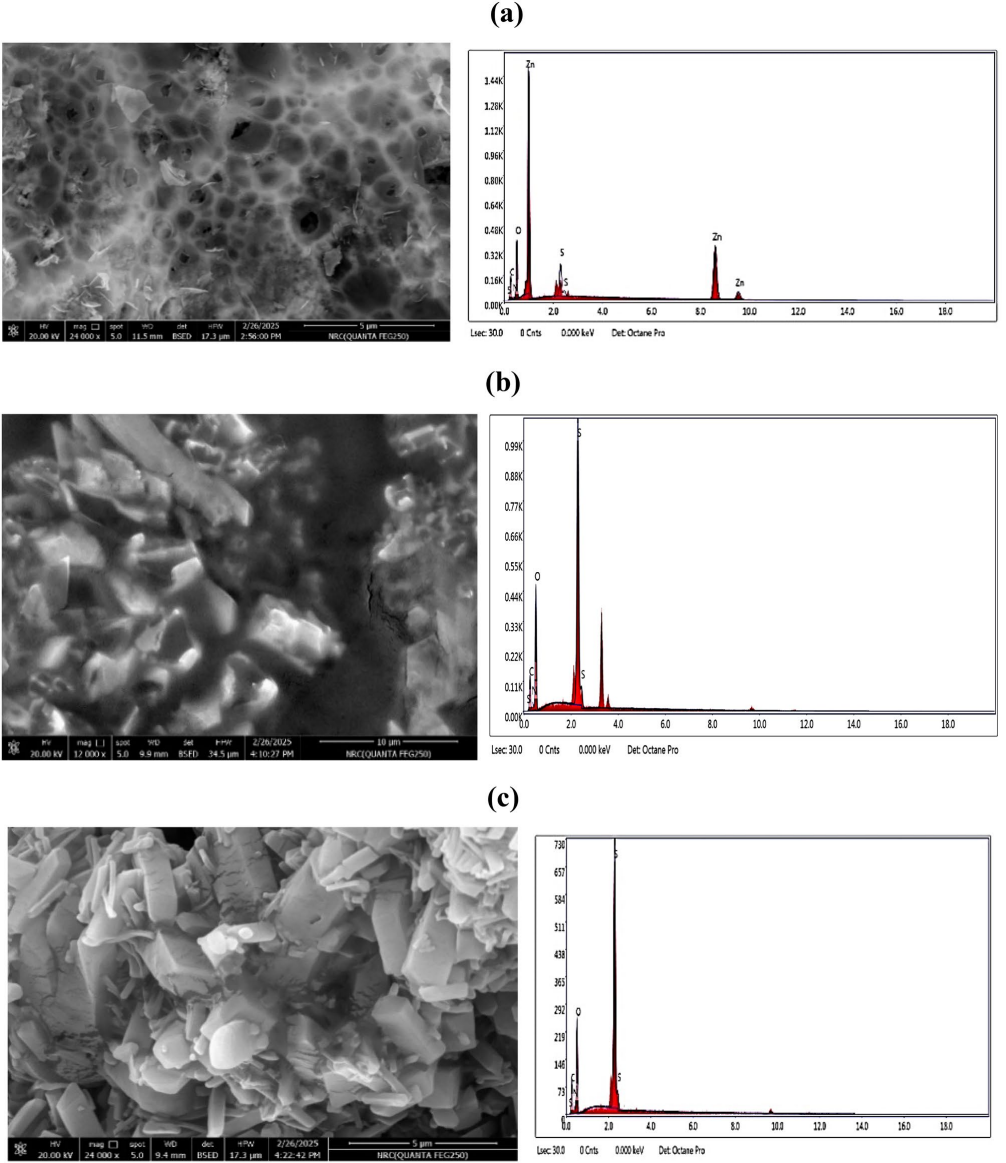
SEM –EDX micrographs of (**a**) ZnO.LPIL, (**b**) CPIL and (**c**) crosslinked AMPS/AA.

The surface morphology of the ZnO.LPIL, CPIL and crosslinked AMPS/AA as well as their chemical analyses were analyzed from SEM and EDEX as represented in Fig. 6a-c, respectively. In this respect, SEM and EDEX image displays ZnO.LPIL (Fig. 6a) clearly shows a porous, sponge-like network with thin, interconnected walls and voids or cavities ranging in size from submicron to a few microns. This morphology is highly indicative of a polymeric structure formed during the capping/stabilization process. The web-like or honeycomb morphology suggests significant templating due to the LPILs. This SEM micrograph reveals a highly porous, interconnected 3D structure of ZnO nanoparticles, stabilized by a sophisticated PIL matrix. The LPIL system likely promotes hydrogen bonding and electrostatic interactions between ZnO surfaces and creating this soft template structure. The EDEX analysis confirm the formation of ZnO and capping with QAMPS/QAA. The SEM images of the CPIL (Fig. 6b) reveals large, angular crystalline structures dispersed throughout the polymeric matrix. These structures suggest a high degree of phase separation, likely due to ionic interactions and the intrinsic rigidity conferred by the sulfonate and acrylate functional groups. The surface texture appears smooth with sharp-edged facets, indicative of a well-ordered microstructure potentially beneficial for controlled release or mechanical reinforcement applications. In contrast, the SEM image of crosslinked AMPS/AA (Fig. 6c) exhibits a markedly different morphology. The SEM image reveals densely packed, irregular crystalline clusters with a rougher surface texture and a more granular appearance. This suggests a more heterogeneous nucleation and growth process during gel formation. The carboxylic and sulfonic acid functionalities of AMPS and acrylic acid may contribute to rapid crosslinking and uneven microstructural development, resulting in the observed morphology. These differences underscore the significant impact of the chemical nature of the crosslinking agents on the resultant microarchitecture of hydrogels. The variation in crystal size, distribution, and surface features is likely to influence not only the mechanical integrity but also the diffusion characteristics and interaction with embedded nanoparticles or therapeutic agents. The EDEX analyses (Fig. 6b **and c**) confirm the quatrenization of AMPS/AA with TEA (Fig. 6b) with increasing the carbon content than non-quaternized AMPA/AA (Fig. 6c).

**Fig. 7:**
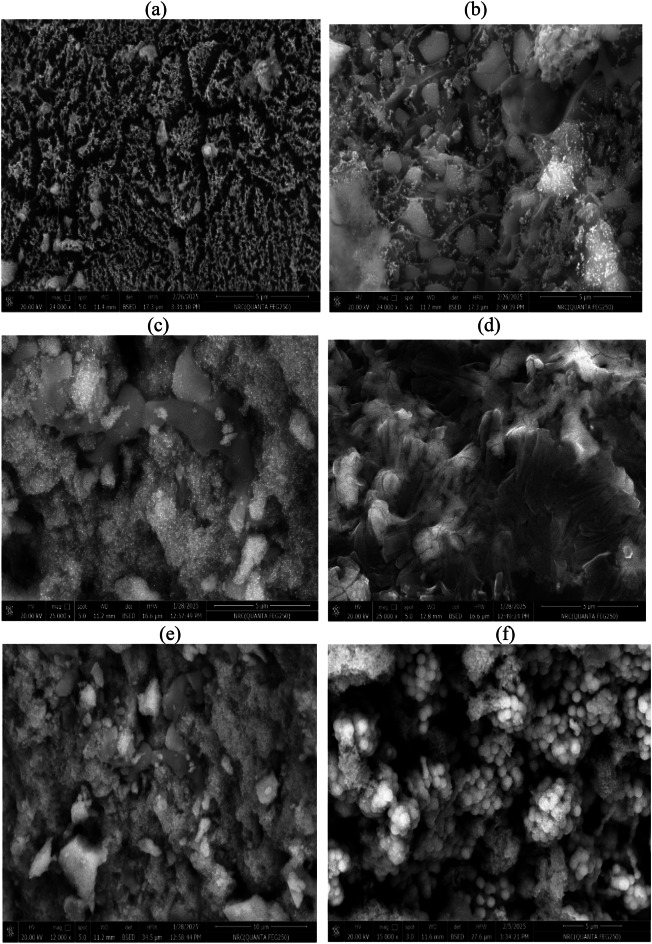
SEM micrographs of (**a**) Fe_3_O_4_.CPIL, (**b**) Fe_3_O_4_.AMPS/AA, (**c**) ZnO (2Wt %).CPIL, (**d**) ZnO (5Wt. %).CPIL, (**e**) Fe_3_O_4_@ZnO(2Wt. %).CPIL and (**f**) Fe_3_O_4_@ZnO (5Wt.%).

The surfaces morphologies of MNCs based on incorporation of Fe_3_O_4_, ZnO and Fe_3_O_4_@ZnO embedded into CPIL and AMPS/AA networks were identified from their SEM micrographs represented in Fig. 7a-f. The SEM micrograph of Fe₃O₄.CPIL (Fig. 7a) confirms that the magnetite nanoparticles are uniformly dispersed and interconnected within the hydrogel matrix. The surface is densely populated with nanoscale features, indicating successful encapsulation and homogeneous distribution. The presence of a porous interconnected network suggests efficient ionic crosslinking, likely mediated by sulfonate and acrylate groups, which strongly coordinate with the surface of magnetite particles. In contrast, the SEM image of Fe₃O₄.AMPS/AA (Fig. 7b) reveals larger, irregularly shaped aggregates with a heterogeneous particle distribution. The hydrogel matrix in this system appears denser and less porous compared to the CPIL-based network, which can be attributed to the steric and ionic influences of AMPS and acrylic acid. These structural differences highlight the significant role of hydrogel composition in governing the dispersion and encapsulation efficiency of magnetite nanoparticles. Figure 7c and d show SEM images of ZnO nanoparticles incorporated into CPIL at 2 and 5 wt%, respectively. At low ZnO loading (2 wt%, Fig. 7c), the matrix displays a smooth and uniform surface with well-dispersed ZnO nanoparticles, suggesting strong compatibility between the polymeric chains and ZnO at low concentrations. The absence of dense bright spots indicates minimal aggregation, while the layered, sheet-like morphology of the CPIL network facilitates uniform embedding of nanoparticles. At higher ZnO loading (5 wt%, Fig. 7d), the surface becomes rougher and more granular, with abundant bright regions corresponding to dense ZnO agglomerates. This clustering suggests that at elevated concentrations, the nanoparticles exceed the stabilization capacity of the CPIL matrix, leading to phase separation, coalescence, and uneven encapsulation. The SEM micrographs in Fig. 7e and f further illustrate Fe₃O₄@ZnO nanocomposites embedded in CPIL with 2 and 5 wt% ZnO, respectively. The images reveal densely packed spherical or near-spherical domains decorated with fine, bright nanoparticle-like features on their surfaces. These features correspond to the formation of zinc ferrite (ZnFe₂O₄) nanoparticles distributed across the hydrogel framework. The uniform embedding and surface decoration confirm successful capping and stabilization by LPILs, which enhance nanoparticle dispersion, suppress agglomeration, and strengthen integration into the CPIL matrix. Furthermore, the triethanolammonium acrylate units provide additional hydrophilicity and flexibility to the polymer network, facilitating strong electrostatic interactions with zinc ferrite. This structural organization highlights the role of LPILs in achieving stable, multifunctional nanocomposites suitable for advanced catalytic and environmental applications.

Overall, the combined XRD, TEM/SAED, and SEM results confirm that the CPIL matrix promotes the formation of highly crystalline ZnFe₂O₄ nanocomposites, with uniform dispersion at lower loadings and controlled aggregation at higher concentrations. The homogeneous embedding and surface decoration highlighted the role of LPILs in nanoparticle capping, which not only prevents agglomeration but also enhances integration into the CPIL network. The triethanolammonium acrylate units further impart hydrophilicity and flexibility, facilitating strong electrostatic interactions with ZnFe₂O₄ and stabilizing the composite architecture. The integration of LPILs ensures enhanced nanoparticle stabilization and matrix compatibility, making these nanocomposites structurally robust and promising for multifunctional applications in catalysis, pollutant remediation, and magnetic separation.

**Fig. 8:**
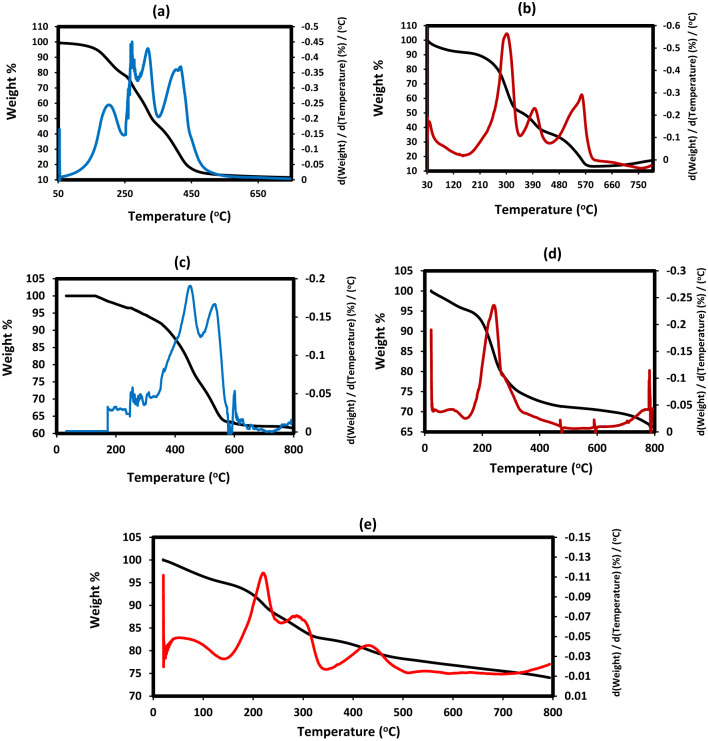
TGA-DTG thermograms of (**a**) LPIL, (**b**) CPIL, (**c**) ZnO.LPIL, (**d**) Fe_3_O_4_.LPIL and (**e**) ZnFe_2_O_4_.LPIL.

**Fig. 9:**
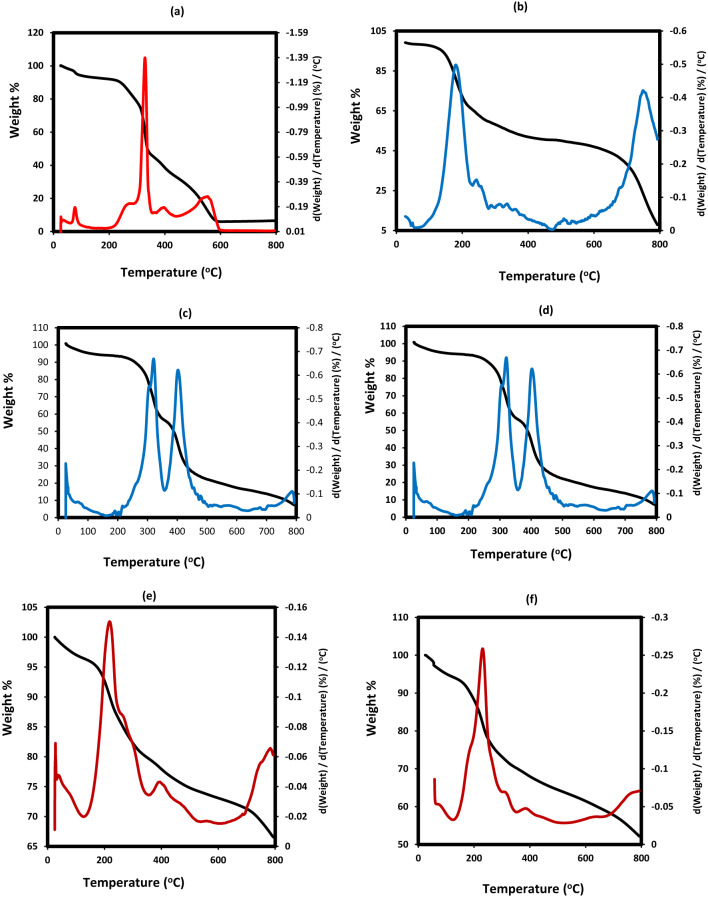
TGA-DTG thermograms of (**a**)ZnO(2%). CPIL, (**b**) Fe_3_O_4_@ZnO(2 Wt.%). CPIL, (**c**) ZnO(5 Wt.%). CPIL, (**d**) Fe_3_O4 @ZnO(5 Wt.%). CPIL, (**e**) Fe_3_O_4_. CPIL and (**f**) ZnFe_2_O_4_.CPIL.

The thermal stability, water, organic and metal oxide contents were determined from TGA-DTG thermograms that summarized for LPIL, CPIL, ZnO.LPIL, Fe_3_O_4_.LPIL and ZnFe_2_O_4_.LPIL in Figs. 8a-e, respectively. TGA-DTG thermograms of ZnO(2%).CPIL, Fe_3_O_4_@ZnO(2 Wt.%).CPIL, ZnO(5 Wt.%).CPIL, Fe_3_O4 @ZnO(5 Wt.%).CPIL, Fe_3_O_4_.CPIL and ZnFe_2_O_4_.CPIL were represented in Fig. 9a-f, respectively. The water content (Wt. %) at degradation temperature below 150 °C, initial degradation temperatures (IDT), and residual contents (Rs %) at degradation temperatures above 650 °C were determined from thermograms and summarized in Table 2. The Rs% were determined at two degradation temperatures 650 and 800 °C and used to evaluate stability of magnetic nanoparticles against oxidation to other iron oxides were determined from the formation of stable weight loss % above 650 °C^41,42^. Moreover, the Rs % in the absence of metal oxides were used to evaluate of the crosslinked carbeneous or graphite residual after degradation of organic compounds. The data listed in Table 2; Figs. 8a-e and 9a-f confirmed the presence of bounded water from water content values ranged from 5 to 10 Wt. % for LPIL, CPIL and their metal oxides to confirm the hydrophilicity of their surfaces to absorb water from humidity. The data confirm that IDT and thermal stability of LPIL increased from 172 to 346 °C with the incorporation of ZnO in their polymer matrices due to surface interactions^43^. the same effect was also observed for CPIL when incorporated with ZnO from 2 to 5% that increased from 205 °C to 241 °C and 270 °C, respectively (Table 1). The presence of iron oxides based on Fe_3_O_4_ or ZnFe_2_O_4_ decreases the IDT of both LPIL and CPIL polymer chains due to their acidic character and oxidation characteristic to increase polymer fragmentation at lower temperature^44^. The thermal stability of magnetic nanoparticles to resist their oxidation to other iron oxides was increased for LPIL than CPIL based on QAMPS/QAA polymer chain to confirm their higher capping efficiency for ZnFe_2_O_4_ and Fe_3_O_4_ NPs with lowering iron oxide contents from 650 to 800 °C (Table 2).

**Fig. 10:**
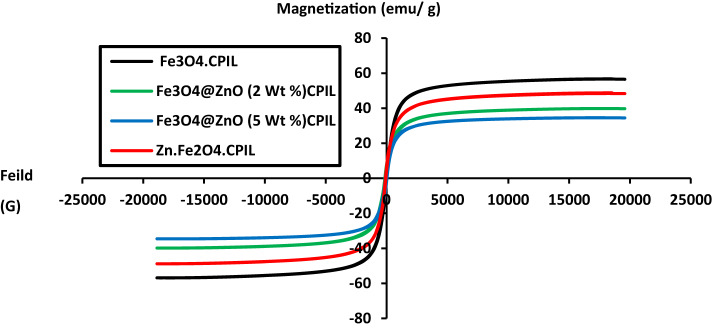
VSM loops of the prepared MNCs at room temperature.

**Table 2:** TGA data of the prepared polymer composites and nanocomposites.

PIL composites	Water content (Wt. %)	IDT (^o^C)	Rs % (Wt. %)		Contents (Wt. %)		Iron oxide contents (Wt %)		
			at 650 °C	at 850 °C			Fe_3_O_4_	ZnFe_2_O_4_	Other iron oxides
LPIL	5	172	12	12	12	-	-	-	-
CPIL	10	205	18	18	18	-	-	-	-
ZnO/LPIL	8	346	62	62	12	50	-	-	-
Fe_3_O_4_/LPIL	7	184	70	67	12	58	55	-	3
Zn.Fe2O4/LPIL	6	167	75	75	12	63	-	63	0
Fe_3_O_4_/CPIL	5	175	72	66	18	54	48	-	6
Zn.Fe_2_O_4_/CPIL	5	155	60	52	18	42	0	34	8
ZnO(2 Wt.%).CPIL	10	241	6.5	6.5	4.5	2	-	-	-
ZnO(5 Wt.%).CPIL	10	270	13	13	8	5	-	-	-
Fe_3_O_4_@ZnO(2 Wt.%).CPIL	5	140	43	10	4.5	38.5	32.5	6	27.5
Fe_3_O_4_@ZnO(5 Wt.%).CPIL	5	200	45	36	8	37	22	15	9

The magnetic properties of the prepared MNCs, including saturation magnetization (Ms), remanent magnetization (Mr), and coercivity (Hc), were evaluated at room temperature from magnetic hysteresis loops recorded using a vibrating sample magnetometer (VSM), as shown in Fig.10. The corresponding values are summarized in Table 3. The samples studied include pure Fe₃O₄.CPIL, Zn-doped Fe₃O₄ (Fe₃O₄@ZnO with 2 wt% and 5 wt% ZnO) within CPIL, and ZnFe₂O₄.CPIL. Among these, pure Fe₃O₄.CPIL exhibits the highest Ms (~ 60 emu/g), confirming its strong magnetic character. In contrast, ZnO incorporation leads to a progressive decrease in Ms, which further diminishes with increasing ZnO content. This decline suggests that ZnO disrupts magnetic ordering by diluting Fe²⁺/Fe³⁺ interactions or by introducing nonmagnetic phases. Similarly, ZnFe₂O₄.CPIL shows a lower Ms compared to pure Fe₃O₄, consistent with the intrinsically weaker magnetic moment of zinc ferrite^45,46^. All samples display narrow hysteresis loops with negligible Mr and Hc, indicative of superparamagnetic or soft magnetic behavior. Such characteristics make these nanocomposites attractive for environmental applications, including adsorption and photocatalysis in water treatment. Overall, the systematic variation in magnetic parameters underscores the tunability of ferrite-based nanocomposites through compositional modification and polymer hybridization.

**Table 3:** Magnetic parameters of the prepared MNCs at room temperature.

PIL composites	Magnetic Properties		
	Ms emu.g^−1^	Mr emu.g^−1^	Hc G
Fe_3_O4.CPIL	56.81	0.67786	7.5259
Zn.Fe2O4.CPIL	48.795	0.45389	6.3980
Fe_3_O_4_@Zno (2 Wt. %).CPIL	34.568	1.6355	25.473
Fe_3_O_4_@Zno (5 Wt. %).CPIL	29.838	0.34521	5.2677

### Application of the prepared MNCs for photodegradation catalysts for MB

**Fig. 11:**
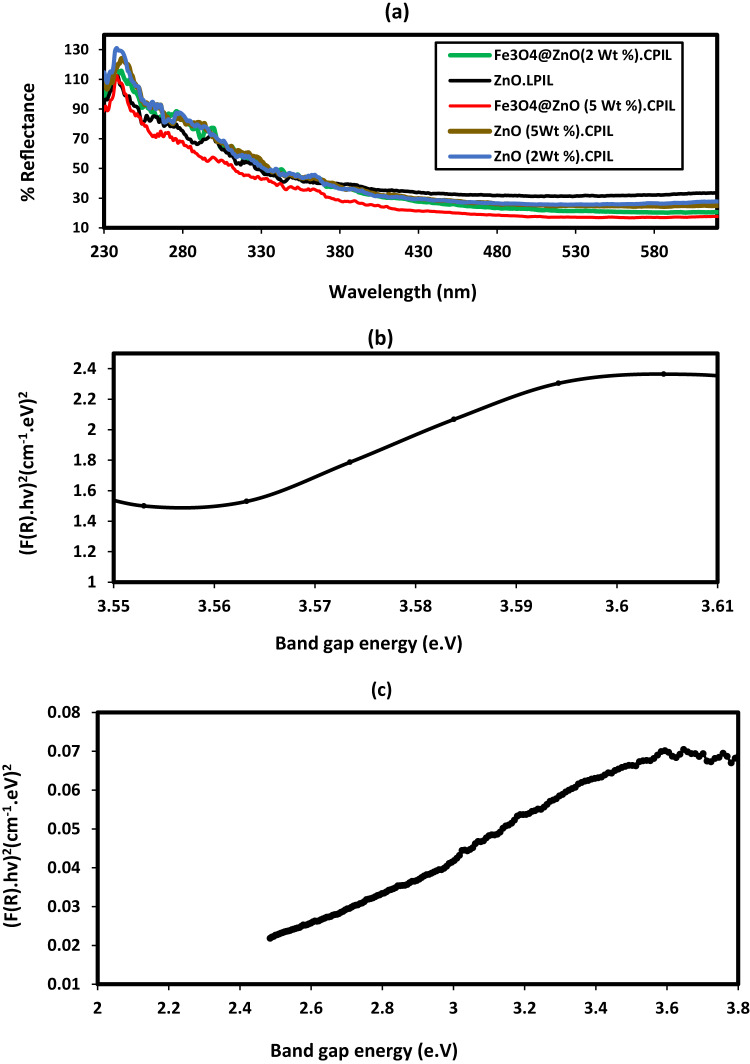
UV-visible spectra of (**a**) Zno and Fe_3_O_4_@ZnO capped with CPIL, the corresponding inset displaying (**b**) Tauc plot of ZnO.LPIL and (**c**) Tauc plot of Fe_3_O_4_@ZnO(5wt.%).CPIL nanocomposites.

The optical properties of ZnO.LPIL, ZnO (2 wt%) CPIL, ZnO (5 wt%) CPIL, Fe₃O₄@ZnO (2 wt%) CPIL, and Fe₃O₄@ZnO (5 wt%) CPIL nanocomposites were evaluated using UV–visible spectroscopy (Fig. 11). The UV–vis spectra (Fig. 11a) display characteristic absorption peaks at 237–240 nm, 280 nm, and 300–330 nm, which can be attributed to PIL, ZnFe₂O₄, and ZnO, respectively. The spinel ferrite absorption is associated with electron excitation from the O-2p valence band to the Fe-3d conduction band^47^. The optical band gap energy (Eg) was estimated from Tauc plots using the equation F(R)hν = A(hν – Eg)², where F(R) is the reflectance coefficient, h is Planck’s constant, ν is photon frequency, and A is a constant (Fig. 11b and c). The reflectance function was calculated as F(R) = (1 – R)²/2R, with R obtained from % reflectance/100. The UV–vis diffuse reflectance spectrum of ZnO.LPIL (Fig. 11b) shows a broad absorption peak at 302 nm, corresponding to a band gap of 3.56 eV. This blue shift compared to bulk ZnO (λ = 376 nm; 3.3 eV) suggests a quantum confinement effect due to the nanoscale particle size^48^. All samples show strong absorption in the UV region, consistent with the wide band gap of ZnO, with a gradual decrease in reflectance at longer wavelengths. Incorporation of Fe₃O₄ into CPIL networks induces a slight red shift of the absorption edge compared to ZnO.LPIL, reflecting enhanced visible-light response due to Fe₃O₄-induced band structure modification and interfacial charge transfer. The Tauc plots (Fig. 11b and c) reveal direct band gaps of 3.56 eV for ZnO.LPIL and 2.35 eV for Fe₃O₄@ZnO (5 wt%) CPIL. The band gap of the synthesized ZnO nanoparticles was estimated at ~ 3.63 eV^49^, while the 2.35 eV value obtained for Fe₃O₄@ZnO.CPIL is in good agreement with reported values for ZnFe₂O₄^48^, though slightly higher than the 1.81 eV reported by Surendra et al.^50^. The reduction in band gap upon Fe₃O₄ incorporation confirms the synergistic role of magnetic core coupling and polymeric functionalization in narrowing the optical gap. This narrowing, together with enhanced visible-light absorption, is expected to promote efficient charge carrier generation, thereby contributing to the improved photocatalytic degradation performance observed in later studies.

**Fig. 12:**
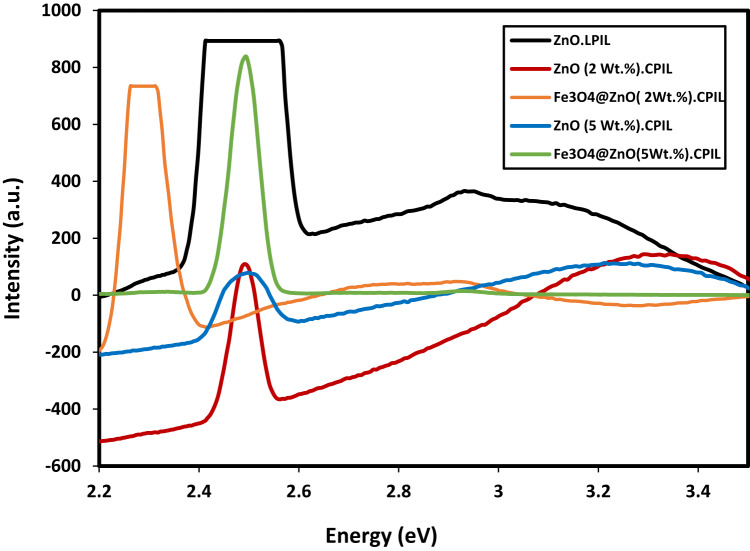
Photoluminescence (PL) properties of ZnO.LPIL, ZnO (2 Wt. %). CPIL, ZnO (5Wt. %). CPIL, Fe_3_O_4_@ZnO (2 Wt. %). CPIL and Fe_3_O_4_@ZnO (2 Wt. %).CPIL.

The photoluminescence (PL) properties of ZnO.LPIL, ZnO (2 wt%) CPIL, ZnO (5 wt%) CPIL, Fe₃O₄@ZnO (2 wt%) CPIL, and Fe₃O₄@ZnO (5 wt%) CPIL were investigated and represented in Fig. 12. PL studies of nanocomposites are of particular interest because of their relevance to photocatalysis, optoelectronics, and sensing technologies. The PL spectra (Fig. 12) reveal emissions in the 2.2–3.4 eV range. Pure ZnO, with its wide band gap (~ 3.37 eV), exhibits a strong near-band-edge UV emission together with visible defect-related emissions. Upon incorporation into polymeric ionic liquid (PIL) networks, the ZnO emission profile is modified, reflecting the influence of surface passivation, polymer–nanoparticle interactions, and reduced defect density. When coupled with ZnFe₂O₄, a spinel ferrite with a narrower band gap (~ 1.9 eV), the resulting Fe₃O₄@ZnO nanocomposites display further modifications in PL behavior. These include partial quenching of defect-related visible emissions, red-shifted emission features, and enhanced charge transfer across the ZnO–ZnFe₂O₄ interface. Such interfacial effects facilitate separation of photoexcited electron–hole pairs and suppress non-radiative recombination, which are beneficial for visible-light-driven photocatalysis. The tunable PL response observed in these nanocomposites highlights the synergistic effects of ZnO, ZnFe₂O₄, and the PIL matrix in tailoring emission properties. This makes them attractive candidates for multifunctional applications including light-emitting devices, photocatalytic degradation, and advanced sensing platforms.

**Fig. 13:**
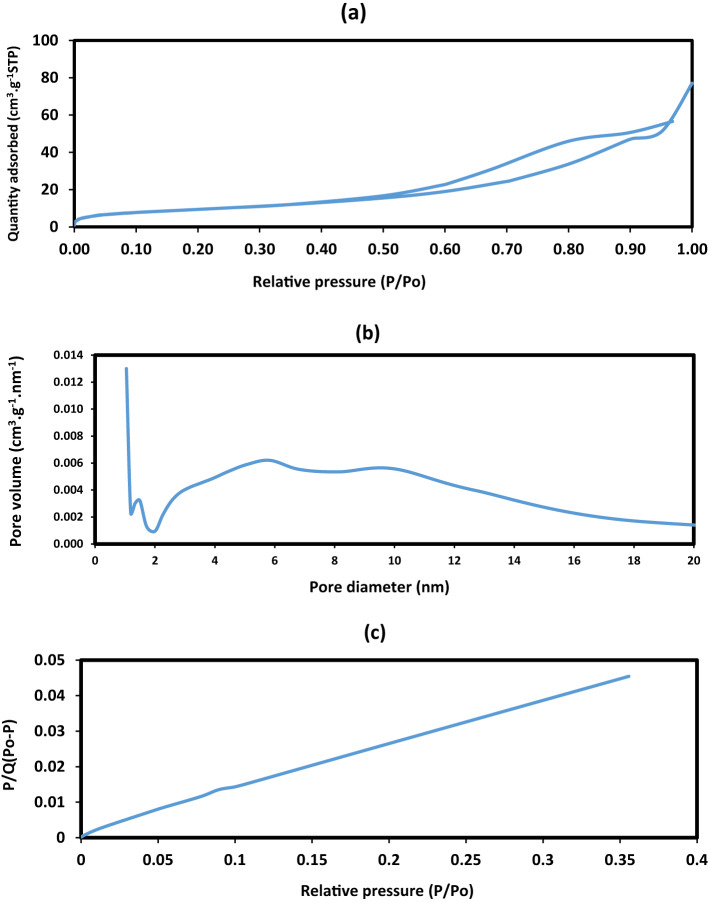
BET curves (**a**) N_2_ adsorption-desorption isotherm, (**b**) pore size distribution hysteresis loop and (**c**) surface area of Fe_3_O_4_@ZnO (2 Wt. %). CPIL.

BET analysis is a critical tool for evaluating the photocatalytic potential of materials, since a larger surface area provides more active sites, which enhances light absorption and charge separation during photodegradation. Furthermore, an optimized pore structure facilitates the diffusion of dye molecules toward active sites, thereby improving overall photocatalytic efficiency^51^. Figure 13a–c presents the Barrett–Joyner–Halenda (BJH) adsorption pore volume, hysteresis loops, and BET surface area of Fe₃O₄@ZnO (2 wt%) CPIL as a representative sample. The isotherm (Fig. 13a) displays the same Type IV(a) profile with an H3 hysteresis loop extending from P/P₀ ≈ 0.45–0.90. Similar Type IV isotherms were observed for all other samples (Figures S1–S4). The N₂ adsorption–desorption isotherm recorded at 77 K exhibits a Type IV(a) profile with a well-defined H3 hysteresis loop extending from ca. P/P₀ ≈ 0.45 to 0.90, which is characteristic of mesoporous solids composed of aggregates of plate-like particles that generate slit-shaped pores. The limited uptake at P/P₀ < 0.10 indicates a minor contribution from microporosity, whereas the steep increase in adsorbed volume at higher relative pressures and the absence of a distinct saturation plateau suggest the presence of wider mesopores and interparticle/macroporous voids. The hysteresis loop and its delayed closure are consistent with capillary condensation–evaporation in open slit pores rather than ink-bottle-type pore blocking. Overall, the pore texture is dominated by mesoporosity, with a secondary contribution from larger voids, in agreement with a hierarchical pore network. The BJH pore size distribution curve (Fig. 13b) shows a pronounced peak in the range of ~ 0–12 nm, indicating a mixture of micro- and mesopores with an approximate 1:5 ratios. The dominance of mesopores is advantageous for catalysis, adsorption, and controlled release applications, as it enhances both molecular diffusion and accessibility to active sites. The BET plot of P/[Q(P₀–P)] versus relative pressure (P/P₀) (Fig. 13c) shows linearity in the range 0.05–0.30, confirming the applicability of the BET equation for this system. From this analysis, the specific surface area, total pore volume, and mean pore diameter of Fe₃O₄@ZnO (2 wt%) CPIL were calculated as 35.40 ± 0.02 m²·g⁻¹, 0.1109 cm³·g⁻¹, and 6.65 nm, respectively. The textural parameters of all synthesized samples are summarized in Table 4. BET analysis of the nanocomposites were represented in the supplementary files Figures S1-S3. BET analysis of the nanocomposites demonstrates pronounced enhancements in textural properties compared with conventional metal oxides. ZnO.LPIL exhibits a modest surface area of 8.73 m²·g⁻¹, which decreases in ZnO (5 wt%) CPIL (7.33 m²·g⁻¹) but increases substantially in Fe₃O₄@ZnO (2 wt%) CPIL (35.40 m²·g⁻¹), accompanied by moderate pore volumes and mesoporous diameters (~ 15 nm). Both ZnO.LPIL and ZnO (5 wt%) CPIL display macropores and mesopores at their surfaces (Figures S1b and S3b), which facilitate the diffusion of adsorbate molecules into the nanocomposite framework. Reported literature values for ZnO vary widely with morphology and synthesis method. For example, commercial ZnO nanoparticles (20 nm) exhibit a surface area of 43.4 m²·g⁻¹, mechanochemically prepared ZnO nanopowder 47.3 m²·g⁻¹, and flower-like ZnO nanostructures 8.8–25.4 m²·g⁻¹; broader reviews report typical ZnO nanostructures spanning 3–50 m²·g⁻¹^52^. In this context, ZnO (5 wt%) CPIL (45 m²·g⁻¹) approaches the upper range of reported ZnO values, while ZnO.LPIL (8.7 m²·g⁻¹) falls at the lower end. ZnFe₂O₄ ferrites also show significant variation: combustion-synthesized ZnFe₂O₄ exhibits 94–116 m²·g⁻¹ depending on the fuel used, sol–gel-derived ZnFe₂O₄ annealed at 500 °C yields ~ 28.7 m²·g⁻¹, while W-substituted ZnFe₂O₄ reaches up to 143 m²·g⁻¹^53^. Compared with these values, Fe₃O₄@ZnO (2 wt%) CPIL (35.4 m²·g⁻¹) falls within or slightly above the mid-range of ZnFe₂O₄ ferrites, surpassing low-annealed ferrites but remaining below combustion- or substitution-enhanced samples. Overall, incorporation of CPIL significantly enhances the surface area and mesoporosity of the composites compared with pristine ZnO or ZnFe₂O₄, with ZnO (5 wt%) CPIL achieving the highest values among the samples. The Fe₃O₄@ZnO composites also show improved textural characteristics, confirming the beneficial role of PIL modification in tailoring surface properties and, consequently, photocatalytic performances.

**Table 4:** BET data of the prepared nanocomposites.

PIL composites	BET data		
	Surface area (m^2^.g^−1^)	Total pore volume (cm^3^.g^−1^)	Average pore diameter (nm)
ZnO.LPIL	8.7311	0.0621	28.426
ZnO (2Wt %).CPIL	27.053	0.1146	14.339
ZnO (5Wt %).CPIL	7.325	0.06079	33.198
Fe_3_O_4_@ZnO(2 Wt %).CPIL	35.404	0.1109	6.650
Fe_3_O_4_@ZnO (5 Wt %).CPIL	28.852	0.1101	15.406

**Fig. 14:**
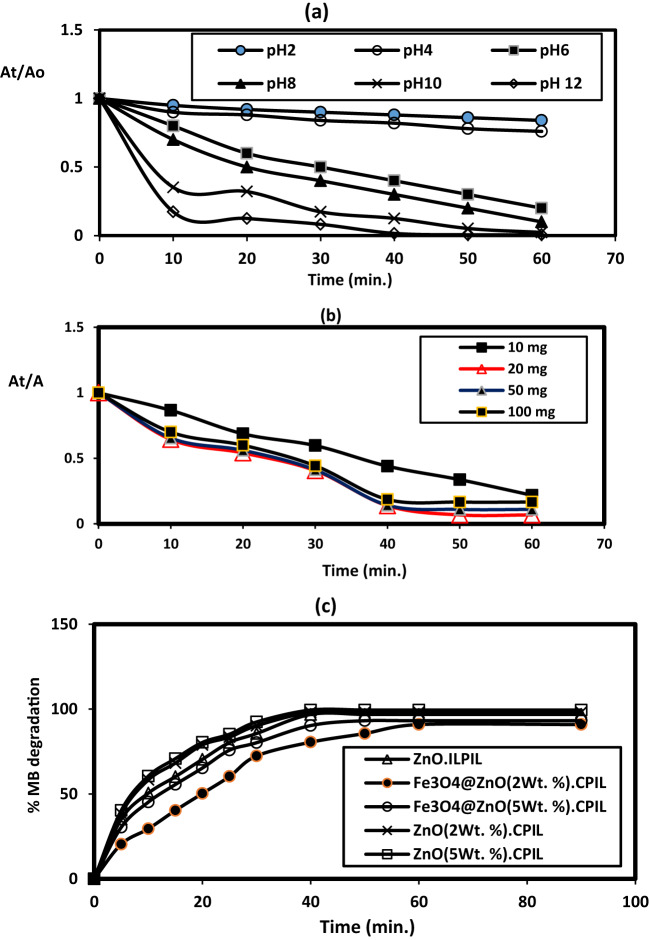
MB Photodegradation parameter in aqueous solution (30 ppm) at different time intervals (**a**) different pHs, (**b**) different catalyst doses and (**c**) % MB degradation using different MNCs at room temperature.

**Fig. 15:**
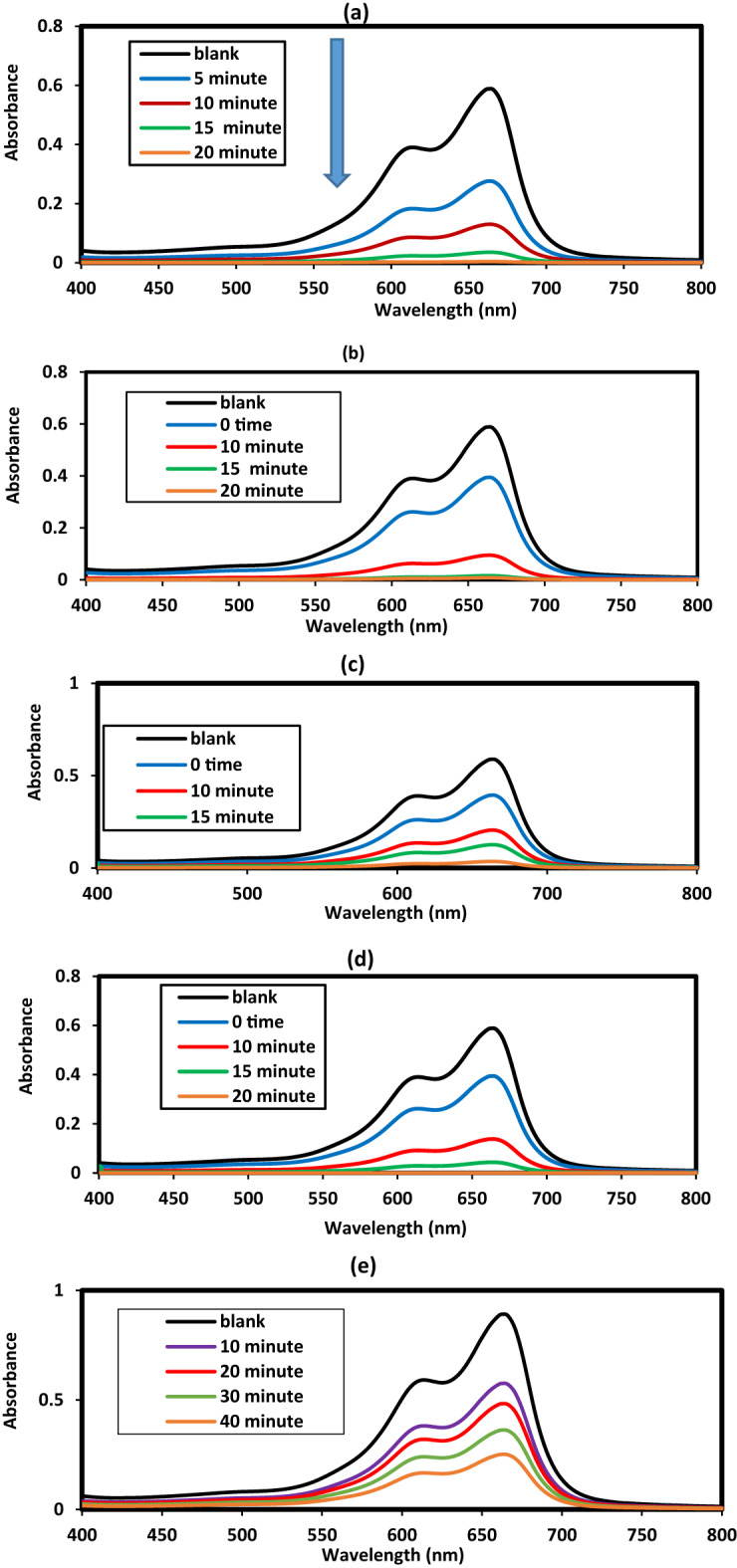
Uv-visible spectra of MB solutions using (**a**) ZnO.LPIL, (**b**) ZnO (2 Wt. %).CPIL, (**c**) Fe_3_O_4_@ZnO (2 Wt. %).CPIL, (**d**) ZnO (5Wt. %).CPIL and (**e**) Fe_3_O_4_@ZnO (5Wt. %). CPIL at optimum conditions.

The development of magnetically separable and highly efficient photocatalysts for wastewater treatment has gained considerable attention in recent years^54–59^. In this work, the photocatalytic activity of hybrid Fe₃O₄@ZnO nanocomposites capped with LPIL and CPIL was investigated for the degradation of MB under UV/visible light irradiation. The synergistic combination of Fe₃O₄, ZnO, and PIL coatings was designed to enhance charge separation, light absorption, and catalytic stability. Photodegradation of MB was carried in an attempt under UV as reported in the experimental section using zinc oxide catalysts such as ZnO.LPIL, ZnO (2 Wt. %).CPIL, ZnO (5Wt. %).CPIL, Fe_3_O_4_@ZnO (2 Wt. %).CPIL and Fe_3_O_4_@ZnO (5 Wt. %).CPIL to obtain optimum conditions. The concentration of MB at 30 mg.L^−1^ was selected as target to degraded although the photodegradation of catalysts efficiency was decreased with increasing dye concentrations due to lowering penetration of UV light on the catalyst surfaces with increasing concentration of MB dye more than 5–20 ppm^54^. Moreover, the adsorption of MB on the catalysts surfaces lowering their efficiency to photodegrade the dye to blocking of active site on the catalyst surfaces^54^. The effects of pH (2–12), ionic strength (0.001 M), catalyst dosage (10–100 mg), and irradiation time on photocatalytic efficiency were systematically investigated, as shown in Fig. 14a–c. Figure 14a illustrates the influence of pH on the absorbance ratio (At/A0) at different irradiation times for 20 mg of ZnO (2 wt%) CPIL dispersed in 100 mL of a 30 ppm MB solution. The results reveal that the degradation rate of MB increased significantly with rising pH, with pH 12 identified as the optimum condition, as it achieved maximum photodegradation in the shortest irradiation time. The enhanced performance at higher pH values is attributed to the increased surface charge potential of both ZnO nanoparticles and CPIL, which promotes adsorption of the positively charged MB molecules and facilitates the transfer of photogenerated charge carriers. This process improves the electronic interactions with the interlayer anions on the photocatalyst surface, thereby enhancing photocatalytic activity^55^. The effect of catalyst dosage was evaluated at pH 12 using Fe₃O₄@ZnO (5 wt%) CPIL (Fig. 14b). Photodegradation efficiency improved markedly when the dosage increased from 10 to 20 mg, but further increases above 20 mg resulted in no significant improvement. The highest MB degradation efficiency was obtained with 20 mg of catalyst in 100 mL of 30 ppm MB solution. This indicates that 20 mg provides an optimal concentration of active sites for generating hydroxyl radicals, which serve as the primary reactive species in dye degradation^29^. At dosages above 20 mg, performance declined slightly due to increased turbidity from the dark ZnO@Fe₃O₄ catalyst, which reduced light penetration during UV irradiation. Thus, 20 mg was identified as the optimal dosage for further experiments. Systematic studies of pH, catalyst dosage, and irradiation time confirmed that pH 12 and 20 mg catalyst dosage provided optimal performance. Increasing the catalyst amount beyond 20 mg led to reduced activity due to increased turbidity and reduced light penetration. The UV–visible spectra of MB photodegradation at different reaction times under optimized conditions (20 mg catalyst dosage, 30 ppm MB, pH 12) for all photocatalysts are summarized in Fig. 15a–e. Figure 14c shows the effect of irradiation time on MB photodegradation efficiency at pH 12 using 20 mg of the prepared catalysts. The results confirm the time-dependent enhancement of photocatalytic activity under the optimized conditions. The order of maximum degradation efficiency was observed as: ZnO (5 wt%) CPIL >ZnO (2 wt%).CPIL >ZnO.LPIL >Fe₃O₄@ZnO (5 wt%).CPIL >Fe₃O₄@ZnO (2 wt%).CPIL, with complete degradation achieved within 40–60 min. Notably, ZnO (5 wt%) CPIL exhibited the highest activity, achieving 99.44% degradation, while Fe₃O₄@ZnO (2 wt%).CPIL achieved 90.9% under the same conditions. These results highlight the superior photocatalytic performance of the prepared catalysts at a relatively low dosage (20 mg) and moderate dye concentration (30 ppm), surpassing previously reported ZnO and ZnFe₂O₄-based photocatalysts^56^. In a significant departure from conventional photocatalyst design, ZnO (5 wt%) CPIL achieved outstanding activity despite its exceptionally low specific surface area of only 7.33 m²·g⁻¹ (Table 4). This contrasts sharply with benchmark photocatalysts such as porous TiO₂, where high BET surface area is typically considered a key performance criterion^53^. The remarkable efficiency of this low-BET material underscores an important insight: the quality and electronic functionality of active sites are more critical than their quantity. The catalyst architecture clearly favors efficient generation, separation, and migration of photogenerated charge carriers over mere pollutant adsorption, resulting in unprecedented photocatalytic quantum yields. XRD analysis further confirmed that incorporation of ZnO nanoparticles into LPIL and CPIL networks strongly influences crystallinity and dispersion. The incorporation of 2 and 5 wt% ZnO into the CPIL matrix (Figs. 2, 3 and 4) resulted in partial retention of ZnO crystallinity, with crystallinity slightly enhanced at higher loading (5 wt%). This structural tuning is crucial for optimizing the optical, electronic, and catalytic properties of ZnO–CPIL hybrids. Collectively, these findings establish a new design paradigm for photocatalytic systems shifting the focus from maximizing surface area to rationally engineering crystallinity, interfacial charge dynamics, and optoelectronic properties for sustainable water treatment.

The Fe₃O₄@ZnO nanocomposites represented a promising platform for MB dye photodegradation as Summarized in Table 5. Figure 14c shows that, under optimium conditions, ZnO (5 wt%) CPIL achieved the highest degradation efficiency (99.44%) within 40–60 min, followed by ZnO (2 wt%) CPIL (97.8%), ZnO.LPIL (94.2%), Fe₃O₄@ZnO (5 wt%) CPIL (92.5%), and Fe₃O₄@ZnO (2 wt%) CPIL (90.9%). When compared with previous reported works (Table 5), the present catalysts demonstrated superior performance. For example, Fe₃O₄/ZnO prepared by solid-state methods degraded only 88.5% of MB (100 mg·L⁻¹) after 2 h^30^, while hydrothermal Fe₃O₄/ZnO achieved 98% under visible light but required higher catalyst dosage (0.4 g·L⁻¹) and longer reaction time^57^. Similarly, microwave-hydrothermal Fe₃O₄/ZnO reached 63% degradation after 1 h under UV^58^. By contrast, the CPIL-modified Fe₃O₄@ZnO nanocomposites in this study achieved up to 99.44% degradation at lower catalyst dosage (20 mg in 100 mL) and moderate dye concentration (30 ppm), outperforming or matching literature results under milder conditions.

**Table 5:** Photo catalytic degradation of MB under UV and visible light in the presence different types of Fe₃O₄@ZnO nanocomposites.

photocatalyst Methods of synthesis	Weight of catalyst g.L^−l^	Concentration of MB g.L^−1^	Time (h)	Degradation %		References
				visible	UV	
Fe_3_O_4_/ZnO Solid state	0.2	100	2	88.5	-	^30^
Fe_3_O_4_/ZnO hydrothermal	0.4	25	2	98	25	^57^
Fe_3_O_4_/ZnO Microwave-hydrothermal	0.5	20	1	-	63	^58^
Fe_3_O_4_/ZnO Co-precipitation	0.2	30	45	71.82	93.16	Present work

It is important to investigate the fitting of kinetic data of MB degradation that usually follows pseudo-first-order kinetics^59^. Pseudo-first-order kinetics is ln(Co/Ct) = k1t where, (k1) is the rate constant in min ^−1^, (t) is time, and (Ct) and (Co) is the final and initial absorbance of the dye, respectively. The values of k1 and a correlation value R^2^ were listed in Table 6, it can be seen that all materials fit the pseudo-first order kinetics with high R ^2^ values close to 1. By comparing the K1 values of the prepared MNCs (Table 6), it was found that from XRD data (Figs. 3 and 4) the higher crystallinity of ZnO (5 Wt. %).CPIL and Fe₃O₄@ZnO (5 Wt. %).CPIL composites reduces electron-hole recombination, boosting UV/visible-light-driven degradation rates^60^. Moreover, the pore structure in the crosslinked network promote MB diffusion and active site accessibility, accelerating dye breakdown kinetics. The efficiency order ZnO (5Wt. %).CPIL >ZnO (2 Wt. %).CPIL >ZnO.LPIL >Fe₃O₄@ZnO (5 Wt. %).CPIL >Fe₃O₄@ZnO (2 Wt. %).CPIL confirms that ZnO generates e⁻/h⁺ pairs, while Fe_3_O_4_ aids charge separation via magnetic interactions and CPIL stabilize radicals (e.g., •OH) and prevent catalyst deactivation. The ZnO (5Wt. %).CPIL exhibits superior porosity and lower particle sizes, as confirmed by TEM/SEM (Figs. 5c and 7d) which enhances MB adsorption capacity and charge carrier mobility, critical for photocatalytic activity.

**Table 6:** The correlation coefficient (R^2^) and kinetics for the photodegradation of MB.

PIL composites	First order kinetics	
	K1(min^−1^)	*R* ^2^
ZnO.LPIL	0.0848	0.9993
ZnO (2Wt %).CPIL	0.0982	0.9923
ZnO (5Wt %).CPIL	0.1256	0.9904
Fe_3_O_4_@ZnO(2 Wt %).CPIL	0.0401	0.992
Fe_3_O_4_@ZnO (5 Wt %).CPIL	0.067	0.9905

**Fig. 16:**
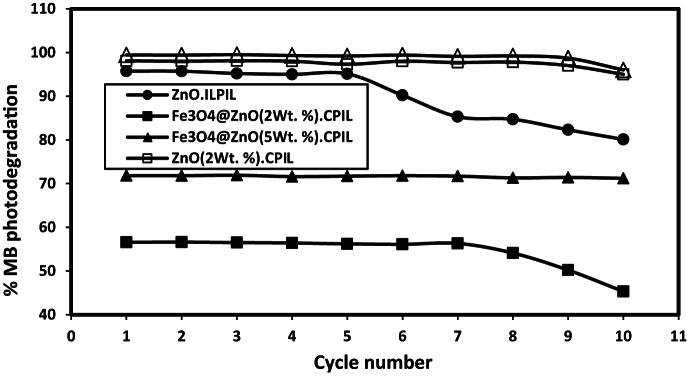
MB photodegradation efficiency after different regeneration cycle numbers at optimium conditions.

**Fig. 17:**
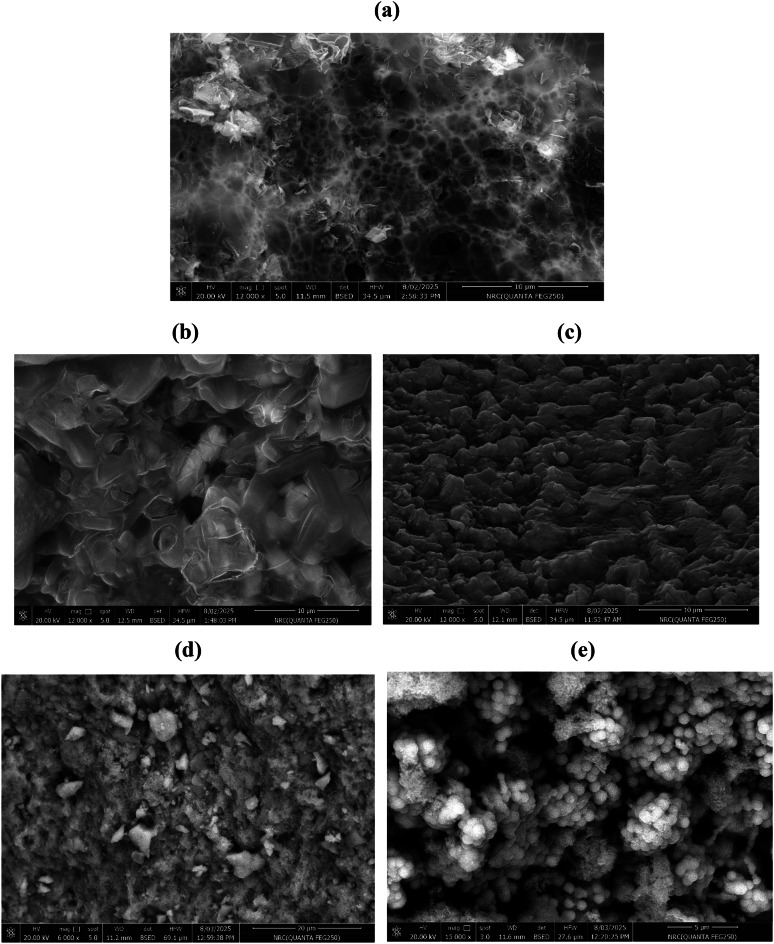
SEM images of (**a**) ZnO.LPIL, (**b**) ZnO (2 Wt. %).CPIL, (**c**) ZnO (5Wt. %).CPIL, (**d**) Fe_3_O_4_@ZnO (2 Wt. %).CPIL and (**e**) Fe_3_O_4_@ZnO (5 Wt. %). CPIL after 5 regeneration photocatalytic cycles.

The catalytic efficiency and catalytic reusability are two important concepts in catalyst sustainability during photocatalysis process. In this respect, the nanocomposites samples were separated by external magnet and centrifuged in order to isolate the photocatalyst followed by washing with de-ionized water three times. After each cycle, the retrieved nanoparticles were transferred into the fresh MB solution. The relation between photocatalytic efficiencies of the prepared catalyst and cycle numbers were represented in Fig. 16. The Fe_3_O_4_@ZnO (5Wt. %)·CPIL maintains its photocatalytic efficiency after 10 cycles of reuse, even after washing with water. This was attributed to superparamagnetic properties which facilitates easy magnetic separation, preventing particle loss during recovery. Moreover, strong covalent bonds in the CPIL matrix protect the ZnO and Fe_3_O_4_ from leaching, unlike ZnO.LPIL which its efficacy reduced after 5 cycles due to weaker interactions. The Fe_3_O_4_@ZnO(2Wt. %)·CPIL shows reduced efficiency after 7 cycles, likely due to lower ZnO loading and less robust protection against leaching. Accordingly, ZnO.LPIL lack a 3D network, leading to gradual ZnO detachment and activity loss. Beyond its initial activity, the catalyst demonstrates exceptional photocatalytic stability and near-perfect reusability across multiple operational cycles. The robust, low-surface-area framework resists the adsorption of recalcitrant intermediate compounds and carbonaceous deposits that typically poison active sites and deactivate conventional catalysts. This combination of supreme degradation efficiency, superior charge carrier dynamics, and outstanding operational durability positions this low-surface-area photocatalyst as a transformative material. Finally, it can be concluding that, the CPIL design combines magnetic recovery of Fe_3_O_4_, chemical stability for ZnO, and optimized porosity, making it ideal for sustainable photocatalysis. The leaching resistance of the prepared nanocomposites after repeated photocatalytic cycles was evaluated by ICP–OES analysis, and the results after the 10th cycle are summarized in Table 7. The data reveal that ZnO.LPIL exhibited the highest Zn leaching, with a concentration of 2.0536 mg L⁻¹ after 10 cycles. This significant release of Zn indicates relatively poor structural stability of ZnO when incorporated only with LPIL. In contrast, the CPIL based composites demonstrated markedly lower leaching values, reflecting improved robustness and resistance to dissolution. For example, ZnO (2 wt%)·CPIL and ZnO(5 wt%)·CPIL showed Zn concentrations of only 0.0005 and 0.0018 mg L⁻¹, respectively, after 10 cycles. Similarly, the magnetite-based hybrids Fe₃O₄@ZnO(2 wt%)·CPIL and Fe₃O₄@ZnO(5 wt%)·CPIL exhibited Zn leaching of 0.0009 and 0.0014 mg L⁻¹, respectively, with Fe levels of 0.0078 and 0.0042 mg L⁻¹. The remarkable reduction in Zn release for CPIL-supported systems compared to ZnO.LPIL highlights the stabilizing role of the crosslinked matrix, which effectively suppresses nanoparticle detachment and metal ion leaching during photocatalytic operation. Moreover, the lower Fe leaching values in magnetite ZnO composites further suggest strong chemical anchoring and synergistic stabilization between ZnO, Fe₃O₄, and CPIL. Overall, the results confirm that CPIL based nanocomposites exhibit excellent reusability and long-term stability, with minimal risk of heavy metal contamination in water after prolonged use, thereby making them more environmentally sustainable for photocatalytic applications. Figure 17a-e shows SEM images illustrating the surface morphology of ZnO.LPIL, ZnO (2Wt %).CPIL, ZnO (5Wt %).CPIL, Fe_3_O_4_@ZnO(2Wt %).CPIL and Fe_3_O_4_@ZnO (5Wt %) after 5 cycles of Photodegradation of MB. Neat ZnO nanoparticles capped with LPIL (Fig. 15a) exhibiting a porous, sponge-like architecture indicative of a loosely network structure same to Fig. 17a. The LPIL provides hydrophilic and ionic domains that may stabilize ZnO but without significant agglomeration. CPIL containing 2 wt% ZnO capped with LPIL (Fig. 17b) shows a denser and more ordered surface with lamellar morphology, suggesting good ZnO dispersion and enhanced network interaction similar to Fig. 7c. This suggests successful capping and interaction with the LPIL, improving the mechanical integrity of the matrix. CPIL with 5 wt% ZnO capped with LPIL (Fig. 17c) displays a rougher surface and localized crystal-like aggregates, indicating the onset of ZnO agglomeration at higher loading similar to Fig. 6d. However, the continuity of the polymer network suggests that ZnO is still mostly integrated within the LPIL matrix, but reaching the limit of stable dispersion. CPIL with 2 wt% ZnO and magnetite nanoparticles (Fig. 17d) revealing a uniform and fine-grained morphology, reflecting homogeneous dispersion and synergistic interaction between ZnO and magnetite within the polymer matrix similar to Fig. 8e. CPIL with 5 wt% ZnO and magnetite nanoparticles (Fig. 17e) have same morphology of similar to Fig. 7f. The structure still appears dense and intact, with spherical agglomerates but no apparent cracks, delamination, or severe surface degradation. Despite repeated cycles of photodegradation, the composite retains its morphology. No signs of ZnO or Fe₃O₄ leaching or phase collapse are visible. The spherical shapes might be due to aggregation under photo exposure but still held tightly in the polymer matrix. This confirms morphological stability and chemical robustness after catalytic cycling. It can be concluding that after 5 consecutive cycles of MB photodegradation, maintaining morphological integrity and surface structure, confirming the high chemical and structural stability of the composite without signs of nanoparticle leaching or matrix degradation.

**Table 7:** The water heavy metal contents after ten cycles using ICP-OES analysis.

PIL composites	Heavy metal content (mg.L^−1^)of water after 1 st cycle		Heavy metal content (mg.L^−1^)of water after 10th cycles	
	Zn	Fe	Zn	Fe
ZnO.LPIL	0.0038	0.021	2.0536	0.0210
ZnO (2Wt %).CPIL	0.0098	0.011	0.0005	0.0082
ZnO (5Wt %).CPIL	0.0013	0.011	0.0018	0.0063
Fe_3_O_4_@ZnO(2 Wt %).CPIL	0.0024	0.011	0.0009	0.0078
Fe_3_O_4_@ZnO (5 Wt %).CPIL	0.0067	0.011	0.0014	0.0042

### Photodegradation mechanism

**Scheme 3:**
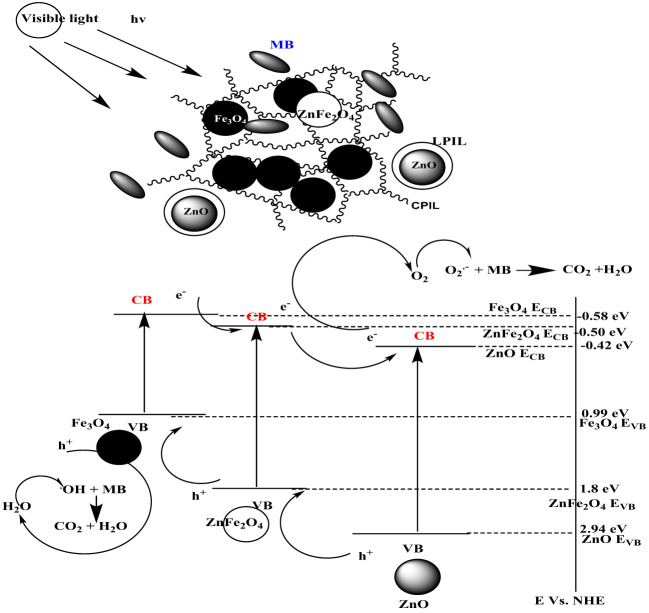
Diagram of Fe₃O₄@ZnO.CPIL photocatalytic activity mechanism.

The proposed degradation mechanism, based on the obtained results and the band structures of magnetite, Fe₃O₄, ZnFe₂O₄, and ZnO, is illustrated in Scheme 3. The incorporation of CPIL enhances organic dye adsorption through electrostatic interactions, facilitates charge transfer across the catalyst surface, and stabilizes the nanocomposite framework under UV and visible irradiation^61^. Upon coupling ZnO, ZnFe₂O₄, and Fe₃O₄, all three semiconductors can absorb photons to generate electron–hole pairs. The conduction and valence band positions of ZnO (–0.45 eV and + 2.94 eV vs. NHE), ZnFe₂O₄ (–0.50 eV and + 1.80 eV), and Fe₃O₄ (–0.58 eV and + 0.99 eV)^54,62^) enable favorable charge migration. Electrons in the conduction bands (CBs) of Fe₃O₄ and ZnFe₂O₄ transfer to the CB of ZnO, while holes generated in the valence band (VB) of Fe₃O₄ remain localized. This spatial separation suppresses electron–hole recombination, thereby enhancing the photocatalytic activity of Fe₃O₄/ZnO compared to pristine ZnO. Subsequently, valence band holes oxidize surface hydroxide ions (OH⁻) or water molecules to produce highly reactive hydroxyl radicals (·OH), which mineralize dye molecules into simpler products^30,54^. In parallel, conduction band electrons are captured by dissolved oxygen to form superoxide radicals (O₂·⁻). The overall process can be summarized as follows as illustrated in Eqs. 3–7:

1. Photoexcitation: Absorption of photons promotes electrons from the VB to the CB of the semiconductor.
2. Hydroxyl radical formation: VB holes oxidize OH⁻/H₂O to generate ·OH radicals.
3. Superoxide radical formation: CB electrons reduce O₂ to O₂·⁻. These reactive oxygen species synergistically drive the degradation of dye molecules into non-toxic products.
  3 $$\text{Photocatalyst}+\text{UV}\Rightarrow\text{e}^-\text{CB}+\text{h}^+\text{VB}$$
  4 $$\text{h}^++\text{H}_2\text{O/OH}^-\Rightarrow\:^.\text{OH}+\text{H}$$
  5 $$\text{e}^-+\text{O}_2\Rightarrow\:^.\text{O}{^2-}$$
  6 $$\text{H}_2\text{O}+\:^.\text{O}^{2-}\Rightarrow{2}\:^.\text{OH}$$
  7 $$\:^.\text{OH}+\text{MB}\Rightarrow\text{CO}_2+\text{H}_2\text{O}$$

The UV light used to excite the catalysts based on ZnO or Fe₃O₄@ZnO (Eq. 3) to produce conduction-band electron (e^−^CB) and a valence-band hole separate (h^+^VB). The holes attack the water surface hydroxyl anion (H_2_O/OH^−^) and yield surface-bound hydroxy radicals (·OH) through oxidation reaction (Eq. 4) that can act as effective centers for photocatalytic reactions. The electrons attack the soluble oxygen in water (Eq. 3) and yields superoxide radical anions (•O^2−^) through reduction reaction that can react with the water surface hydrogen cation (H_2_O/H^+^) to produce also ·OH that can act as effective centers for MB photocatalytic reactions degradation. The concentrations of MB are effective in its photo degradation and it believed to be destroyed through direct oxidation by the ·OH radicals. PL spectra of ZnO, ZnFe_2_O_4_ and Fe_3_O_4_.ZnO catalysts **(**Fig. 12**)** were used to evaluate the (e⁻/h⁺) recombination rate on their photodegradation properties. Generally, it is well known that a higher and lesser PL emission intensity confirm a rapid and a deliberate (e⁻/h⁺) recombination rate, respectively. The pristine ZnO/LPIL exhibits a moderate near-band-edge (NBE) emission around 3.2 eV, corresponding to the recombination of free exactions in the ZnO wurtzite lattice. A broad visible emission between 2.2 and 2.8 eV is also observed, typically assigned to deep-level emissions (DLE) originating from oxygen vacancies (VO), zinc interstitials (ZnI), and related intrinsic defects^63^. For ZnO (2 Wt.%).CPIL, the NBE intensity decreases and a redshift in the emission maximum is noted, suggesting enhanced defect-related recombination due to increased polymer–nanoparticle interactions. The Fe₃O₄@ZnO (2 wt%) CPIL sample exhibits a strong, narrow peak in the visible region (~ 2.4 eV), indicating dominant DLE processes facilitated by interfacial charge transfer between Fe₃O₄ and ZnO. Increasing the ZnO loading to 5 wt% in CPIL results in an intensified NBE peak and reduced visible emission, implying improved crystallinity and suppressed defect density. In contrast, Fe₃O₄@ZnO (5 wt%) CPIL presents a sharp, high-intensity emission in both NBE and DLE regions, reflecting synergistic enhancement of radiative recombination through Fe₃O₄–ZnO interfacial effects. These results confirm that Fe₃O₄ incorporation and CPIL capping modulate the ZnO defect landscape and exciting recombination pathways, which can have a significant influence on photocatalytic performance. High-surface-area materials often suffer from poor crystallinity and a high density of crystalline defects and grain boundaries, which act as efficient recombination centers for photogenerated electrons (e⁻) and holes (h⁺). In contrast, ZnO.LPIL, ZnO (2 Wt.%).CPIL and ZnO.(5Wt. %).CPIL catalyst’s highly crystalline structure minimizes these recombination losses, ensuring a maximal flux of charge carriers to the surface where redox reactions occur. Furthermore, its optimized band gap and superior light-harvesting efficiency ensure a greater utilization of incident photons. For the degradation of large molecular dyes like MB, the material’s open surface structure, free from microporous confinement, facilitates enhanced mass transport and more efficient interactions between the active oxidative species (e.g., •OH, O₂•⁻) and the target pollutant, preventing pore blockage and intermediate fouling.

**Fig. 18:**
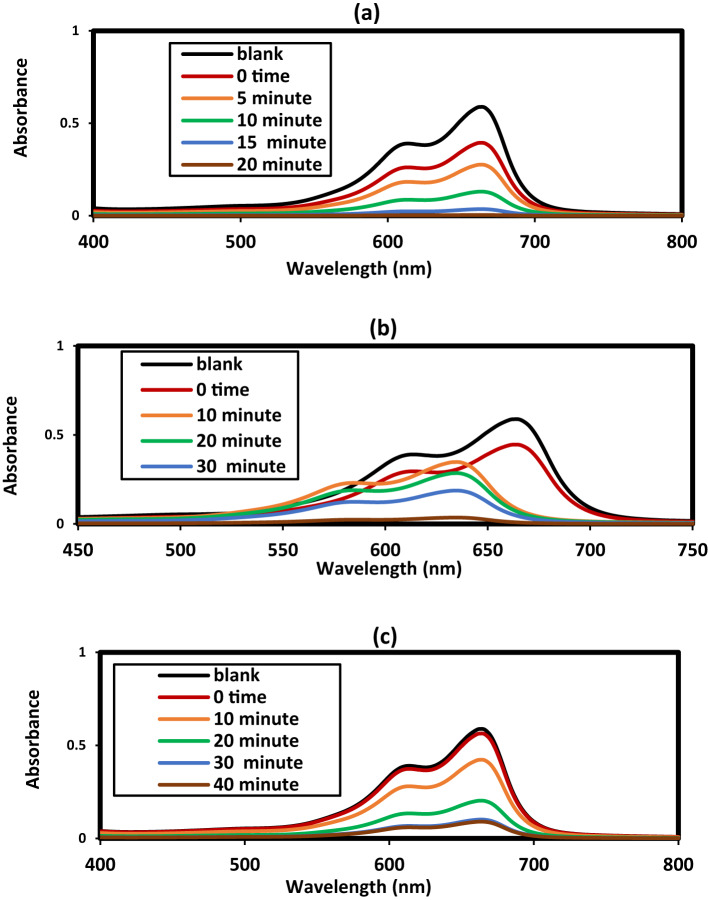
Uv-visible spectra of MB solutions in the presence of ZnO.LPIL using (**a**) isopropanol, (**b**) EDTA, and (**c**) AgNO3 as ROS at optimum conditions.

The effect of ROS based on isopropanol, EDTA and AgNO_3_ added to MB aqueous solutions on the catalysts degradation efficiencies was summarized in Fig. 17 to investigate the reaction mechanism for the prepared catalysts under visible light as represented in the experimental section. Their MB Uv- visible spectra were recorded at different times and represented in Fig. 18a-c for ZnO.LPIL as representative and Figures S5-8 for other prepared nanocomposites.

**Fig. 19:**
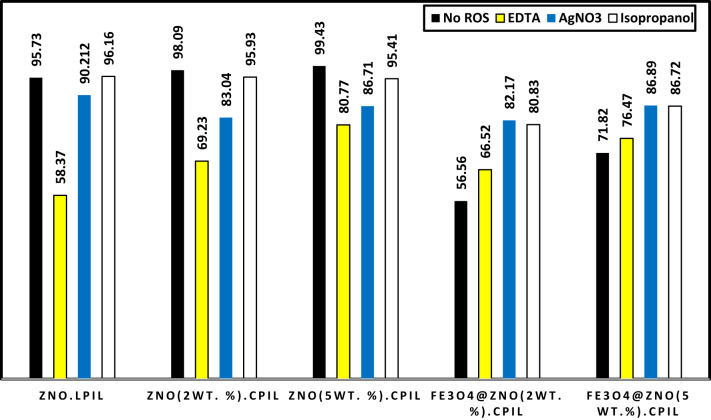
Effect of ROS on the MB photocatalytic degradation efficiency of the prepared catalysts.

It was noticed from Fig. 19 that the addition of isopropanol the photocatalytic system enhances the degradation efficiency of MB dye due to its role as a scavenger for holes (h⁺) or hydroxyl radicals (•OH), which reduces electron-hole (e⁻/h⁺) recombination and promotes the generation of reactive oxygen species (ROS) such as superoxide radicals (•O₂⁻). EDTA (as an electron scavenger) reduces the formation of •O₂⁻ by trapping (e⁻), slowing down the reaction in all catalytic systems based on ZnO but slightly increases the MB photo degradation efficiency in case of Fe₃O₄@ZnO. When AgNO₃ is added, it captures free electrons (e⁻) to form metallic silver (Ag⁰), slightly reduces MB photo degradation efficiency in case of ZnO because the electrons are no longer available for •O^2−^ formation. The MB photo degradation efficiency was slightly increased when AgNO_3_ added to Fe₃O₄@ZnO system. This can be attributed to the ability of Fe₃O₄/ZnO/ZnFe₂O₄ nanocomposites to convert metallic silver (Ag⁰) to Plasmonic Ag nanoparticles to form Ag/ZnO, Ag/Fe_3_O_4_ and Ag/ZnFe_2_O_4_ that improved charge separation (Schhottky barrier formation) which prevents to reduce e⁻/h⁺ recombination^64,65^. The incorporation of Fe₃O₄ beside ZnO provides superparamagnetic properties as discussed previously for easy catalyst recovery as well as extends light absorption into the visible range (~ 2.2 eV bandgap) and facilitates electron transfer to reduce e⁻/h⁺ recombination. The presence of ZnO acts as a UV-active photocatalyst (bandgap ~ 3.3 eV), generates electron-hole pairs under irradiation, producing reactive oxygen species (ROS: •OH, •O₂⁻). The presence of CPIL enhances dye adsorption via electrostatic interactions, improves charge transport across the catalyst surface and stabilizes the nanocomposite structure. These •OH, •O₂⁻radicals are the ones that then degrade the pollutant to form safer materials such as water and carbon dioxide. Pseudo-first-order kinetics is anticipated, as ROS concentrations remain steady-state during irradiation.

## Conclusion

Magnetic ZnO nanocomposites were prepared at low temperature without calcination at higher temperature using linear and crosslinked PIL as capping to control their surfaces morphology, sizes, thermal stability, magnetic properties and photocatalytic degradation efficacies. The XRD data elucidate the crystalline ZnO.LPIL NPs were obtained and were weakly crystalline or well-dispersed at 2 Wt.% within the CPIL network. Moreover, the increasing of ZnO.LPIL content up to 5 Wt. % improved ZnO crystalline ordering at higher loading. This may be due to ZnO aggregation or domain formation at higher content, partially recovering its intrinsic crystalline structure that responsible on the formation of porous nanocomposites. The incorporation of magnetite using in-situ technique to form Fe_3_O_4_@ZnO.CPIL reveals that at 2% ZnO loading, a well-defined crystalline ferrite phase forms, while at 5% ZnO, crystallinity diminishes, possibly due to excessive ZnO disrupting ferrite crystallization or forming amorphous zinc-rich domains. The crystal sizes of ZnO.LPIL, Fe_3_O_4_@ ZnO (2 Wt %)CPIL, and Fe_3_O_4_@ ZnO (5 Wt. %)CPIL are 42.4 nm, 70.1 nm, and 52.6 nm, respectively. Moreover, the d-spacing values of ZnO.LPIL, Fe_3_O_4_@ ZnO (2 Wt %)CPIL, and Fe_3_O_4_@ZnO (5 Wt %)CPIL and Fe_3_O_4_.CPIL are 2.47715 Å, 2.73175 Å, and 2.73327 Å, respectively. TEM and SEM data clearly shows a spherical nanoparticles capped with thin porous, sponge-like network, interconnected walls and voids or cavities ranging in size from submicron to a few microns. The web-like or honeycomb morphology suggests significant templating pf ZnO and Fe3O4@ZnO with LPIL and CPIL. UV–vis spectra showed the maximum absorbance peak at around 330–350 nm, which confirmed the formation of ZnO, ZnFe_2_O_4_ and Fe_3_O_4_.ZnO nanocomposites. The optical energy bandgap of the synthesized nanoparticles was determined to be 2.32–3.36 eV. From the optical absorption analysis, the nanocomposites of ZnO, ZnFe_2_O_4_ and Fe_3_O_4_.ZnO were discovered to be in the ultraviolet range with green emission.The order for reaching the maximum MB photodegradation efficiency are arranged in the order ZnO (5Wt. %).CPIL > ZnO (2 Wt. %).CPIL > ZnO.LPIL > Fe₃O₄@ZnO (5 Wt. %).CPIL > Fe₃O₄@ZnO (2 Wt. %).CPIL that riches the maximum efficiency during time from 40 to 60 min. The high degradation efficiency of the prepared catalyst of ZnO (5Wt. %).CPIL to Fe₃O₄@ZnO (2 Wt. %).CPIL from 99.44 to 56.56% in short time elucidates the higher photocatalytic efficiency were obtained at lower 20 mg catalyst dosage and MB concentration 30 ppm. The nanocomposites were highly stable, and the degradation was due to electrons. This work has shown that it is possible to produce an environmentally friendly photocatalyst at low temperature that can be used in both the textile industry for wastewater treatment.

## Supplementary Information

Below is the link to the electronic supplementary material.

Supplementary Material 1

## Data Availability

Data availabilityThe datasets generated and analyzed during the current study are available from the corresponding author upon reasonable request.Code availabilityNot applicable for that section.
