# Supplementary material for: Superparamagnetic Fe₃O₄/ZnO/ZnFe₂O₄ nanocomposites for efficient photocatalytic degradation of methylene blue from water under UV light
Source: Sci Rep. 2025 Nov 18;15:40393. doi: 10.1038/s41598-025-24533-3 (PMC12627692; doi:10.1038/s41598-025-24533-3)
Supplement: Supplementary file 1 — Supplementary Material 1 [file 41598_2025_24533_MOESM1_ESM.docx]

**Superparamagnetic Fe₃O₄/ZnO/ZnFe₂O₄ Nanocomposites for Efficient Photocatalytic Degradation of Methylene Blue from water under UV light**

By

Alia.A.Melegy^a^, Yasser k. Abdel-Monem^b^, Farag. A. Ali^b^, Nermine E. Maysour^a^, and Ayman M. Atta^a*^

^a^ Petroleum application department, Egyption petroleum research institute, Nasr city, Cairo11727, Egypt

^b^ Menoufia University, Faculty of Science, Chemistry Department, Egypt

^*^Corresponding author: E-mail [aatta@epri.sci.eg](mailto:aatta@epri.sci.eg); [Khaled_00atta@yahoo.com](mailto:Khaled_00atta@yahoo.com)

Table S1. XRD diffraction peaks and their Miller indices (hkl) of ZnO.LPIL (Ref. Code zinc oxide 04-015-5830).

| Pos. [°2θ] | d-spacing [Å] | Height [cts] | FWHM Left [°2θ] | Rel. Int. [%] | h | k | l | Area [cps*°2θ] | Area [cts*°2θ] |
| --- | --- | --- | --- | --- | --- | --- | --- | --- | --- |
| 31.7921 | 2.81474 | 51.39 | 0.3149 | 51.57 | 1 | 0 | 0 | 26.60 | 15.96 |
| 34.4938 | 2.60021 | 47.48 | 0.3149 | 47.64 | 0 | 0 | 2 | 24.58 | 14.75 |
| 36.2655 | 2.47715 | 99.65 | 0.1968 | 100.00 | 1 | 0 | 1 | 32.24 | 19.35 |
| 47.5461 | 1.91245 | 19.49 | 0.3936 | 19.56 | 1 | 0 | 2 | 12.61 | 7.57 |
| 56.5885 | 1.62645 | 26.03 | 0.3936 | 26.12 | 1 | 1 | 0 | 16.85 | 10.11 |
| 62.7772 | 1.48018 | 24.98 | 0.3149 | 25.06 | 1 | 0 | 3 | 12.93 | 7.76 |
| 67.8870 | 1.38068 | 21.70 | 0.3542 | 21.78 | 1 | 1 | 2 | 12.64 | 7.58 |
| 69.0723 | 1.35986 | 9.98 | 0.5117 | 10.01 | 2 | 0 | 1 | 8.39 | 5.04 |

Table S2. XRD diffraction peaks and their Miller indices (hkl) of ZnFe_2_O_4_.CPIL.

| Pos. [°2θ] | d-spacing [Å] | Height [cts] | FWHM Left [°2θ] | Rel. Int. [%] | h | k | l | Area [cps*°2θ] | Area [cts*°2θ] |
| --- | --- | --- | --- | --- | --- | --- | --- | --- | --- |
| 30.0465 | 2.97417 | 4.61 | 0.7872 | 7.86 | 2 | 2 | 0 | 5.96 | 3.58 |
| 32.3700 | 2.76580 | 58.65 | 0.0590 | 100.00 | 3 | 1 | 1 | 5.69 | 3.42 |
| 35.3174 | 2.54144 | 17.90 | 0.4330 | 30.53 | 2 | 2 | 2 | 12.75 | 7.65 |
| 43.0145 | 2.10283 | 2.80 | 0.9446 | 4.78 | 4 | 0 | 0 | 4.35 | 2.61 |
| 46.5569 | 1.95075 | 2.68 | 0.4723 | 4.57 |  |  |  | 2.08 | 1.25 |
| 53.4828 | 1.71333 | 2.18 | 0.5510 | 3.72 |  |  |  | 1.98 | 1.19 |
| 56.8638 | 1.61923 | 4.70 | 0.6298 | 8.01 |  |  |  | 4.86 | 2.92 |
| 58.0174 | 1.58975 | 6.67 | 0.2362 | 11.38 | 4 | 2 | 2 | 2.59 | 1.55 |
| 63.8424 | 1.37613 | 1.89 | 0.3149 | 3.22 | 4 | 4 | 0 | 1.67 | 1.00 |

Table S3. XRD diffraction peaks and their Miller indices (hkl) of ZnO(2 Wt. %).CPIL.

| Pos. [°2θ] | d-spacing [Å] | Height [cts] | FWHM Left [°2θ] | Rel. Int. [%] | h | k | l | Area [cps*°2θ] | Area [cts*°2θ] |
| --- | --- | --- | --- | --- | --- | --- | --- | --- | --- |
| 12.0911 | 7.32000 | 15.53 | 0.2558 | 39.58 |  |  |  | 6.53 | 3.92 |
| 18.1380 | 4.89100 | 28.77 | 0.3149 | 73.33 |  |  |  | 14.89 | 8.94 |
| 19.0906 | 4.64903 | 13.67 | 0.2165 | 34.85 |  |  |  | 4.87 | 2.92 |
| 20.4596 | 4.34094 | 14.45 | 0.2362 | 36.83 |  |  |  | 5.61 | 3.37 |
| 21.5936 | 4.11548 | 15.02 | 0.2755 | 38.29 |  |  |  | 6.81 | 4.08 |
| 23.8813 | 3.72616 | 39.23 | 0.2165 | 100.00 |  |  |  | 13.96 | 8.38 |
| 26.2904 | 3.38993 | 28.25 | 0.2558 | 72.00 |  |  |  | 11.88 | 7.13 |
| 27.4086 | 3.25412 | 10.63 | 0.2165 | 27.09 |  |  |  | 3.78 | 2.27 |
| 32.1691 | 2.78261 | 11.68 | 0.3936 | 29.77 |  |  |  | 7.56 | 4.54 |
| 42.1009 | 2.14632 | 5.52 | 0.6298 | 14.08 |  |  |  | 5.72 | 3.43 |
| 60.2739 | 1.53552 | 3.07 | 0.6298 | 7.81 |  |  |  | 3.17 | 1.90 |

Table S4. XRD diffraction peaks and their Miller indices (hkl) of Fe_3_O_4_@ZnO (5 Wt, %).CPIL

| Pos. [°2θ] | d-spacing [Å] | Height [cts] | FWHM Left [°2θ] | Rel. Int. [%] | h | k | l | Area [cps*°2θ] | Area [cts*°2θ] |
| --- | --- | --- | --- | --- | --- | --- | --- | --- | --- |
| 28.2360 | 3.16062 | 13.01 | 0.1771 | 35.35 | 0 | 0 | 2 | 3.79 | 2.27 |
| 30.3257 | 2.94742 | 7.07 | 0.4723 | 19.21 | 2 | 2 | 0 | 5.49 | 3.29 |
| 32.7847 | 2.73175 | 36.79 | 0.1181 | 100.00 | 3 | 1 | 1 | 7.14 | 4.29 |
| 35.6930 | 2.51556 | 23.88 | 0.4723 | 64.89 | 1 | 0 | 1 | 18.54 | 11.12 |
| 40.3041 | 2.23776 | 7.93 | 0.3149 | 21.57 | 4 | 0 | 0 | 4.11 | 2.46 |
| 43.3786 | 2.08602 | 6.31 | 0.4723 | 17.16 |  |  |  | 4.90 | 2.94 |
| 47.0139 | 1.93285 | 3.89 | 0.2362 | 10.58 | 1 | 0 | 2 | 1.67 | 1.00 |
| 57.3517 | 1.60661 | 9.03 | 0.6298 | 24.54 |  |  |  | 9.35 | 5.61 |
| 58.4228 | 1.57968 | 5.58 | 0.4723 | 15.16 | 1 | 0 | 3 | 4.33 | 2.60 |
| 62.9350 | 1.47685 | 10.64 | 0.5510 | 28.92 | 4 | 4 | 0 | 9.64 | 5.78 |

Table S5. XRD diffraction peaks and their Miller indices (hkl) of Fe_3_O_4_@ZnO (5 Wt, %).CPIL.

| Pos. [°2θ] | d-spacing [Å] | Height [cts] | FWHM Left [°2θ] | Rel. Int. [%] | h | k | l | Area [cps*°2θ] | Area [cts*°2θ] |
| --- | --- | --- | --- | --- | --- | --- | --- | --- | --- |
| 23.0458 | 3.85932 | 10.67 | 0.1968 | 10.55 | 1 | 0 | 0 | 3.45 | 2.07 |
| 28.1702 | 3.16785 | 10.08 | 0.2165 | 9.96 | 0 | 0 | 2 | 3.59 | 2.15 |
| 30.3026 | 2.94961 | 5.97 | 0.5510 | 5.91 | 2 | 2 | 0 | 5.41 | 3.25 |
| 32.7659 | 2.73327 | 101.12 | 0.1574 | 100.00 | 3 | 1 | 1 | 26.17 | 15.70 |
| 35.6353 | 2.51949 | 19.17 | 0.6298 | 18.96 | 1 | 0 | 1 | 19.85 | 11.91 |
| 40.2459 | 2.24086 | 7.45 | 0.3542 | 7.36 | 4 | 0 | 0 | 4.34 | 2.60 |
| 46.9350 | 1.93591 | 10.05 | 0.1574 | 9.94 | 1 | 0 | 2 | 2.60 | 1.56 |
| 49.7388 | 1.83316 | 1.51 | 0.7872 | 1.49 |  |  |  | 1.95 | 1.17 |
| 52.8768 | 1.73153 | 3.90 | 0.2952 | 3.85 | 4 | 2 | 2 | 1.89 | 1.13 |
| 57.3277 | 1.60722 | 4.42 | 0.6298 | 4.37 |  |  |  | 4.57 | 2.74 |
| 58.4098 | 1.58000 | 14.13 | 0.2952 | 13.98 | 1 | 0 | 3 | 6.86 | 4.12 |
| 63.0172 | 1.47512 | 8.82 | 0.6298 | 8.72 | 4 | 4 | 0 | 9.13 | 5.48 |
| 65.5464 | 1.36900 | 4.19 | 0.3149 | 4.14 | 2 | 2 | 0 | 2.17 | 1.30 |
| 67.0514 | 1.22435 | 3.27 | 0.4723 | 3.23 | 1 | 1 | 2 | 2.54 | 1.52 |

Table S6. XRD diffraction peaks and their Miller indices (hkl) of Fe_3_O_4_.CPIL.

| Pos. [°2θ] | d-spacing [Å] | Height [cts] | FWHM Left [°2θ] | Rel. Int. [%] | h | k | l | Area [cps*°2θ] | Area [cts*°2θ] |
| --- | --- | --- | --- | --- | --- | --- | --- | --- | --- |
| 30.0465 | 2.97417 | 4.61 | 0.7872 | 7.86 |  |  |  | 5.96 | 3.58 |
| 32.3700 | 2.54144 | 17.90 | 0.4330 | 30.53 | 2 | 2 | 0 | 5.69 | 3.42 |
| 35.3174 | 2.76580 | 58.65 | 0.0590 | 100.00 | 3 | 1 | 1 | 12.75 | 7.65 |
| 43.0145 | 2.10283 | 2.80 | 0.9446 | 4.78 | 4 | 0 | 0 | 4.35 | 2.61 |
| 53.4828 | 1.71333 | 2.18 | 0.5510 | 3.72 | 4 | 2 | 2 | 1.98 | 1.19 |
| 56.8638 | 1.61923 | 4.70 | 0.6298 | 8.01 | 5 | 1 | 1 | 4.86 | 2.92 |
| 63.0174 | 1.58975 | 6.67 | 0.2362 | 11.38 | 4 | 4 | 0 | 2.59 | 1.55 |
| 74.6424 | 1.37613 | 1.89 | 0.3149 | 3.22 | 5 | 3 | 3 | 1.67 | 1.00 |

**(a)**


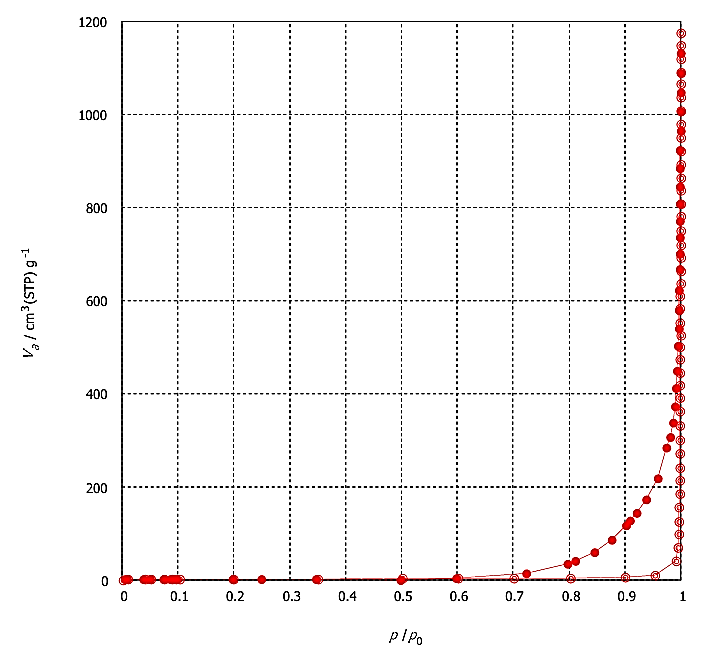


Figure S1. BET curves a) N_2_ adsorption-desorption isotherm, and b) pore size distribution hysteresis loop of ZnO.LPIL.

Figure S2. BET curves a) N_2_ adsorption-desorption isotherm, and b) pore size distribution hysteresis loop of ZnO (2 Wt. %).CPIL.

**(a)**


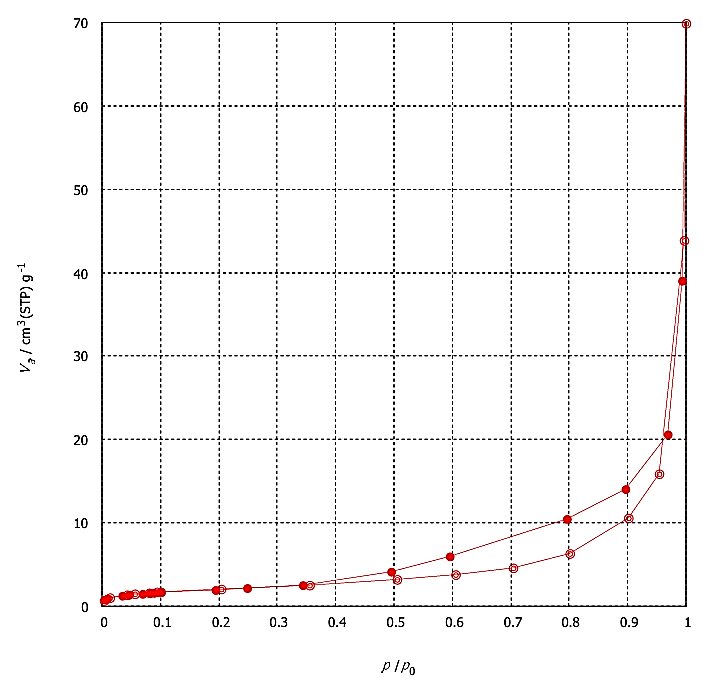


Figure S3. BET curves a) N_2_ adsorption-desorption isotherm, and b) pore size distribution hysteresis loop of ZnO (5 Wt. %).CPIL.

Figure S4. BET curves a) N_2_ adsorption-desorption isotherm, and b) pore size distribution hysteresis loop of Fe_3_O_4_@ZnO (5 Wt. %).CPIL.

Figure S5. Uv-visible spectra of MB solutions in the presence of ZnO (2 Wt. %).CPIL using a) isopropanol, b) EDTA, and c) AgNO3 as ROS at optimum conditions.

Figure S6. Uv-visible spectra of MB solutions in the presence of Fe_3_O_4_@ZnO (2 Wt. %).CPIL using a) isopropanol, b) EDTA, and c) AgNO3 as ROS at optimum conditions.

Figure S7. Uv-visible spectra of MB solutions in the presence of ZnO (5Wt. %).CPIL using a) isopropanol, b) EDTA, and c) AgNO3 as ROS at optimum conditions.

Figure S8. Uv-visible spectra of MB solutions in the presence of Fe_3_O_4_@ZnO (5 Wt. %).CPIL using a) isopropanol, b) EDTA, and c) AgNO3 as ROS at optimum conditions.
